# Influence of Cholesterol on the Insertion and Interaction of SARS-CoV‑2 Proteins with Lipid Membranes

**DOI:** 10.1021/acsabm.5c00776

**Published:** 2025-06-06

**Authors:** Priscila S. Ferreira, Barbara B. Gerbelli, Jorge Cantero, Federico Iribarne, Ana C. H. de Castro-Kochi, Leandro T. Kochi, Fabiola L. Castro, Wendel A. Alves

**Affiliations:** † Center for Natural and Human Sciences, 74362 Federal University of ABC, Santo André 09210-580, Brazil; ‡ Theoretical Chemical Physics and Biology Group, Mathematics-DETEMA Department, Faculty of Chemistry, UdelaR, General Flores 2124, Montevideo 11800, Uruguay

**Keywords:** dipalmitoylphosphatidylcholine, SARS-CoV-2, lipid membranes, cholesterol dynamics, Langmuir monolayer, small angle X-ray scattering, atomic force microscopy, molecular dynamics simulations

## Abstract

Cholesterol is an essential sterol in cell membranes that regulates organization and fluidity. This biomolecule has been identified as one of the critical factors in the internalization process of various viruses in human cells. Therefore, understanding these mechanisms is crucial for a deeper comprehension of viral pathogenicity in the search for practical therapeutic approaches against viral diseases. The biochemical and biophysical processes related to these diseases are highly complex. For this reason, studying model systems capable of mimicking the interaction of lipid membranes with cholesterol and proteins is fundamental. In this work, we propose to study the structural and elastic changes in mono-, bi-, and tridimensional lipid systems composed of dipalmitoylphosphatidylcholine (PC) with varying amounts of cholesterol in the presence and absence of the S protein (Spike) and its receptor-binding domain (RBD) from SARS-CoV-2. To characterize these systems, we used both experimental and theoretical approaches such as Langmuir trough, atomic force microscopy (AFM), small-angle X-ray scattering (SAXS), electrochemical methods, and molecular dynamics (MD) simulations. With the interpretation of all results obtained in this work, it was possible to propose a structural model of the membrane in the presence of cholesterol and the interaction with the Spike protein and RBD. The behavior of the adsorption isotherm and SAXS data, together with the results provided by MD simulations, led us to conclude that cholesterol in PC monolayers promotes local alterations, inducing the formation of more rigid membrane regions. More importantly, cholesterol plays a crucial role in facilitating the allocation of SARS-CoV-2 proteins in lipid systems. This is especially true for the Spike protein, which displayed a non-ACE2 mediated stable binding to the lipid membrane with high internalization.

## Graphical abstract

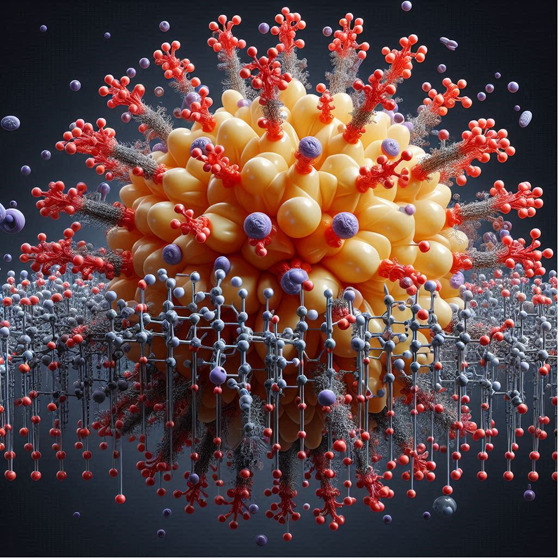

## Introduction

Cholesterol (Chol) is the predominant sterol molecule in cell membranes^1^ and plays a crucial role in regulating their organization and fluidity.^2^ An increase in membrane rigidity is observed when cholesterol is incorporated into phospholipid bilayers. This leads to greater order in lipid chains and results in liquid-ordered (*L*_0_) – liquid-disordered (*L*_α_) phase separation, attributed to alterations in the organization of lipid carbon chains near cholesterol molecules.^3^ Interestingly, the manifestation of these phases can be modulated by the quantity of cholesterol introduced to the lipid membranes.^4^ Furthermore, these specific domains in cell membranes are instrumental in mediating the adhesion of various proteins,^5–7^ underpinning several cellular mechanisms.^8^

Preclinical studies indicate that cell membrane cholesterol can influence innate and adaptive immunity in patients.^9,10^ This suggests that it is possible to modulate the maturation and function of immune cells by affecting cell surface receptors through cholesterol levels. As a result, cholesterol’s presence becomes highly pertinent to viral internalization and replication.^11^ Moreover, cholesterol might enhance the incorporation of the virus into the host’s body.^4,12,13^

In 2019, SARS-CoV-2 emerged as the causative agent of coronavirus disease 2019 (COVID-19). Rapidly spreading globally, this viral outbreak was declared a public health emergency of international concern.^14,15^ The disease presents a broad spectrum of clinical manifestations, ranging from asymptomatic carriers to individuals needing intensive care. Notably, the most severe cases (accounting for 20% of patients) had underlying chronic comorbidities such as high blood pressure, diabetes mellitus as well as kidney and heart diseases.^16^ Remarkably, cholesterol levels are higher in these conditions than in healthy individuals.^17–19^

It is well recognized that SARS-CoV-2 S protein (Spike protein) has a key role in the infection of human cells by mediating the fusion of viral and cellular membranes. The main step of the virus entrance process entails the binding of the S1 subunit of the Spike protein, more precisely the Receptor Binding Domain (RBD), to the angiotensin-converting enzyme 2 (ACE2) receptor present on the host cell surface. Notwithstanding this mechanism, an unexpected discovery by Asandei et al. revealed that the S1 subunit of the Spike protein readily permeabilized lipid bilayer membranes devoid of the ACE2 receptor in a mechanically driven phenomenon.^20^

Deepening our understanding of viral mechanisms is paramount when seeking practical therapeutic approaches against viral diseases. The biochemical and biophysical intricacies associated with these diseases are immensely complex.^21,22^ As such, studying model systems that emulate the interaction between lipid membranes and proteins has become vital.^23–25^ While the role of cholesterol in regulating membrane organization and fluidity has been extensively studied, fewer reports focus on how cholesterol-rich membranes specifically interact with viral proteins, such as those from SARS-CoV-2. This gap in the literature, particularly regarding the structural and mechanical parameters of these systems in two-dimensional and three-dimensional models, highlights the need for further investigation.^26^

Herein, we studied the structural and physicochemical properties of lipid systems using a cellular model composed of the lipid dipalmitoyl-phosphatidylcholine (PC).^27–29^ We varied the molar ratios of cholesterol to PC ([Chol/PC]) to investigate how cholesterol incorporation into lipid membranes might influence protein adhesion. Specifically, we selected the Spike protein and RBD domain from SARS-CoV-2 for analysis. We aimed to discern the impact of protein size and distinct hydrophobic regions on the intercalation of these biomolecules within cholesterol-rich lipid membranes.

We began our study with small-angle X-ray scattering (SAXS) to investigate the structural characteristics and gain insights into the self-assembly mechanisms of multilamellar vesicles, both in the absence and presence of proteins. In parallel, we extensively analyzed cholesterol-doped lipid monolayers to examine how varying cholesterol concentrations affect the adsorption isotherm, compressibility modulus, and surface potential (SPOT) and how introducing proteins alters these physicochemical properties. Atomic force microscopy (AFM) was employed to observe potential structural modifications at the nanoscale. To further elucidate the molecular interactions underlying our experimental observations, we performed molecular dynamics (MD) simulations on PC monolayers with the Spike protein and RBD at different [Chol:PC] ratios. Our results underline the significant role of cholesterol in modulating the properties of lipid membranes and facilitating the mechanical attachment and penetration of the SARS-CoV-2 Spike protein in both lipid monolayers and bilayers.

## Materials and Methods

### General Information

The membrane composition was based on 1,2-dipalmitoyl-*sn*-glycero-3-phosphatidylcholine (PC) (≤99%) with a molecular weight (MPC) of 734 g mol^–1^ and cholesterol (≤99%) with a molecular weight (MChol) of 386.65 g mol^–1^. Both were sourced from Sigma-Aldrich. The recombinant SARS-CoV-2 Spike protein used in our experiments was sourced in a ready-to-use form from the Cell Culture Engineering Laboratory (LECC) from UFRJ, prepared under conditions that ensure its stability and functionality. According to the Certificate of Analysis, the protein was in a buffer containing 50 mmol L^–1^ biotin, 3 mmol L^–1^ NaN_3_, pH 7.4. It was stored at conditions (4 °C and −20 to −80 °C) that have been demonstrated to maintain its stability. The protein preparation follows the protocols and recommendations described by Wrapp et al.,^30^ ensuring a precise quantification for our experimental use with a concentration measured by Nanodrop to be 0.215 mg mL^–1^. The SARS-CoV-2 RBD was procured from BioLinker company, which reported a purity of 98% and a molecular weight (MRBD) of 38,000 g mol^–1^. Spike and RBD were solubilized in ultrapure water (type 2) at 1 to 0.1 mg mL^–1^. Stock solutions for PC and cholesterol were prepared by solubilizing them in chloroform to achieve a final concentration of 1 mg mL^–1^. PC and cholesterol utilized in this study were obtained from Sigma-Aldrich and were used as received without additional purification.

### Polyacrylamide Gel Electrophoresis (SDS-PAGE)

The purity and molecular weight of Spike and RBD recombinant proteins were assessed using 10% SDS-PAGE under denaturing conditions. Samples were prepared in a denaturing buffer (62.5 mM Tris-HCl, pH 6.8, 10% glycerol, 2% SDS, 5% β-mercaptoethanol, and 0.01% bromophenol blue) and heated at 95 °C for 10 min for denaturation. The denatured samples were then loaded onto the gel, and electrophoresis was performed at 160 V for 60 min using a Tris-Glycine-SDS running buffer. After separation, proteins were visualized by staining the gel with Coomassie blue. The gel showed a band of ∼ 180 kDa for the Spike protein and ∼ 38 kDa for the RBD protein, consistent with their expected molecular weights,^31^ as shown in Figure S1 in the Supporting Information.

### Small Angle X-ray Scattering (SAXS)

SAXS experiments of lipid vesicles, both in the absence and presence of varying protein concentrations, were conducted at the bioSAXS B21 beamline, Diamond Light Source, U.K. We maintained a [Chol/PC] ratio of 0.25 for these experiments while adjusting protein concentrations between 0.2 mg mL^–1^ and 0.2 ng mL^–1^.

The lipid and protein solutions were loaded onto a 96-well plate of the EMBL BioSAXS robot. These solutions were then automatically injected through an automated sample changer into a quartz capillary with an internal diameter of 1.8 mm, where they were exposed to the X-ray beam. Approximately ten frames were acquired for each sample with a continuous sample flow through the capillary. The B21 beamline operated with a sample holder-detector distance set at 3.9 m and a wavelength (λ) of 1.00 Å. Image collection was facilitated using a Pilatus 2 M detector. Subsequent data processing tasks, including background subtraction and radial averaging, were carried out using the dedicated ScÅtter software for the beamline.^32^

### Langmuir Trough Experiments

To explore the interaction of proteins with different monolayer compositions and examine the adsorption isotherm, experiments were executed in a Langmuir trough (KSV Instruments) at a constant temperature of 21 °C. The trough’s subphase was consistently composed of 190 mL of Milli-Q water. Different [Chol/PC] ratios ranging from 0.10 to 0.42 were prepared and spread on the subphase’s surface. After applying, a 15 min wait ensured the complete evaporation of the organic solvents. Six compression and decompression cycles were executed for each experiment at a steady speed of 15 mm min^–1^. The Spike protein and RBD were introduced into the aqueous subphase when the lateral pressure in the monolayers was constant at 30 mN/m. 50 μL of protein stock solutions was added to the Langmuir trough subphase.

Consequently, the proteins’ final concentrations in the aqueous subphase were set at 20 ng mL^–1^ and 0.2 μg mL^–1^. To stabilize the monolayer with the proteins present in the subphase, a 30 min waiting period was observed. Postwaiting, another set of six compression and decompression cycles was conducted. Supplementary surface potential measurements were carried out utilizing the KSV NIMA SPOT setup.

### Atomic Force Microscopy (AFM)

For AFM measurements of monolayers, we used the Langmuir–Blodgett (LB) method to transfer the lipid monolayer onto a mica substrate. This approach involves collecting the film vertically, ensuring the nonpolar section is oriented upward. AFM analyses were implemented with the MultiMode VIII from Bruker’s NanoScope V series at the National Nanotechnology Laboratory in Campinas, Brazil.

The experiments operated in tapping mode, employing a silicon tip with a specified force constant of 2.8 N m^–1^ and a resonant frequency of roughly 75 kHz. Scans were collected over 512 × 512 pixels, covering areas between 0.5 and 2.0 μm^2^. Subsequently, both topological and phase data were processed and interpreted using the Gwyddion software package.^33^

### Electrochemical Characterization

Electrochemical investigations used indium tin oxide (ITO) as the conductive substrate. We adopted the Langmuir–Schaefer (LS) method to transfer the monolayer onto the substrate. This technique focuses on the horizontal collection of the film, with emphasis on positioning the polar part of the film upward.

To modify the ITO electrode with the monolayer, 2 μL of an 8 μg mL^–1^ antiRBD antibody was added, both in the presence and absence of proteins. A waiting period of 20 min was observed to guarantee the complete drying of the antibody solution.

Electrochemical readings were carried out using the Metrohm Autolab PGSTAT 302N system, equipped with FRA2, and operated using the NOVA 2.1.3 software. A conventional three-electrode system was in place: the ITO electrode modified with the lipid monolayer functioned as the working electrode, a platinum wire served as the counter electrode, and a silver/silver chloride electrode (Ag/AgCl (3 mol L^–1^ KCl)) acted as the reference against which all potentials were measured.

The assays were performed in a 0.1 mol L^–1^ KCl solution (pH 7.3) that contained 5 mmol L^–1^ of the redox probe K_4_Fe­(CN)_6_/K_3_Fe­(CN)_6_. Electrochemical impedance spectroscopy measurements occurred in the electrolyte solution at the half-wave potential *E*_1/2_ derived from prior cyclic voltammetry (around 240 mV), spanning a frequency range of 0.1 Hz to 30 kHz.

### Molecular Dynamics Simulations

An initial lipid monolayer was constructed with a base composition of PC on a surface of 100 × 100 Å, consisting of a total of 160 PC units for the complexes with RBD, and on a surface of 210 × 210 Å with a total of 635 PC units for the complexes with the Spike protein. Monolayers containing cholesterol were produced by uniformly replacing PC units across the surface to yield 10% and 30% molar cholesterol concentrations. The surface of the monolayers and their components were calculated and built using the CHARMM-GUI interface.^34^ Fully glycosylated RBD (PDB Id: 6ZLR) and SARS-CoV-2 Spike Protein (PDB Id: 6VSB) data (coordinates and protein structure files) were obtained from the COVID-19 Proteins Library deposited in the CHARMM archive.^35^ Of note here is that the three-dimensional structure of the Spike protein lacks the complete transmembrane domain (TM) and cytoplasmatic tail (CT). The initial positioning of RBD and Spike was calculated using PPM (Positioning of Proteins in Membranes) software,^36^ which identified the optimal orientations within the membranes. A thin cavity was made in the membrane around the suggested protein position to avoid steric conflicts. Next, localized geometry optimizations occurred to improve local site coupling. After that, systems were solvated in a rectangular box using the TIP3P model for water molecules. Lipid monolayers were deposited on a water film with an air (vacuum) interface in the *x*–*y* plane on both lipid tails, as described in a previous protocol.^37^ The box size in the *z*-direction was set to 250 and 700 Å for RBD and Spike, respectively, to avoid unscreened electrostatic interactions through the vacuum. The starting lipid monolayer setup, which is common to all simulation runs, can be appreciated in Figure S2. Simulation boxes for the complexes with RBD comprised over 110,000 atoms, while the number of atoms in the Spike protein boxes exceeded 900,000. MD simulations were undertaken with the NAMD 3.01 package,^38^ running in GPU resident mode, and the CHARMM36 force field.^39^ Trajectories were generated under periodic boundary conditions and a canonical ensemble (NVT) at a constant temperature of 310° K.

The integration time step was 2 fs, the nonbonded cutoff radius was set to 12 Å, and the long-range electrostatic interactions were described utilizing the PME algorithm. Six cycles of minimization and relaxation were carried out to ensure correct equilibration of the initial boxes, during which constraints were gradually reduced until vanishment. The ensuing production stage proceeded without restrictions for 200 ns, with a systematic sampling every 10 ps. The analysis phase of the simulations was assisted with VMD software^40^ and ad hoc Python scripts, in particular, the molecular visualization and processing of trajectories. To measure the thickness of the lipid monolayers and the penetration of proteins into them, eqs S4 and S5 (see Support Information) were applied.

## Results and Discussion

### Small Angle X-ray Scattering

SAXS experiments were conducted on model membranes composed of PC, both in the presence (Figure 1B,D) and absence (Figure 1A,C) of cholesterol. We maintained a [Chol/PC] ratio of 0.25 to explore how proteins might alter the lipid membrane structure. A wide range of protein concentrations was utilized, ranging from ng mL^–1^ to mg mL^–1^. SDS-PAGE analysis confirmed the purity and integrity of the Spike and RBD proteins used in this and all subsequent experiments (Figure S1).

**Figure 1:**
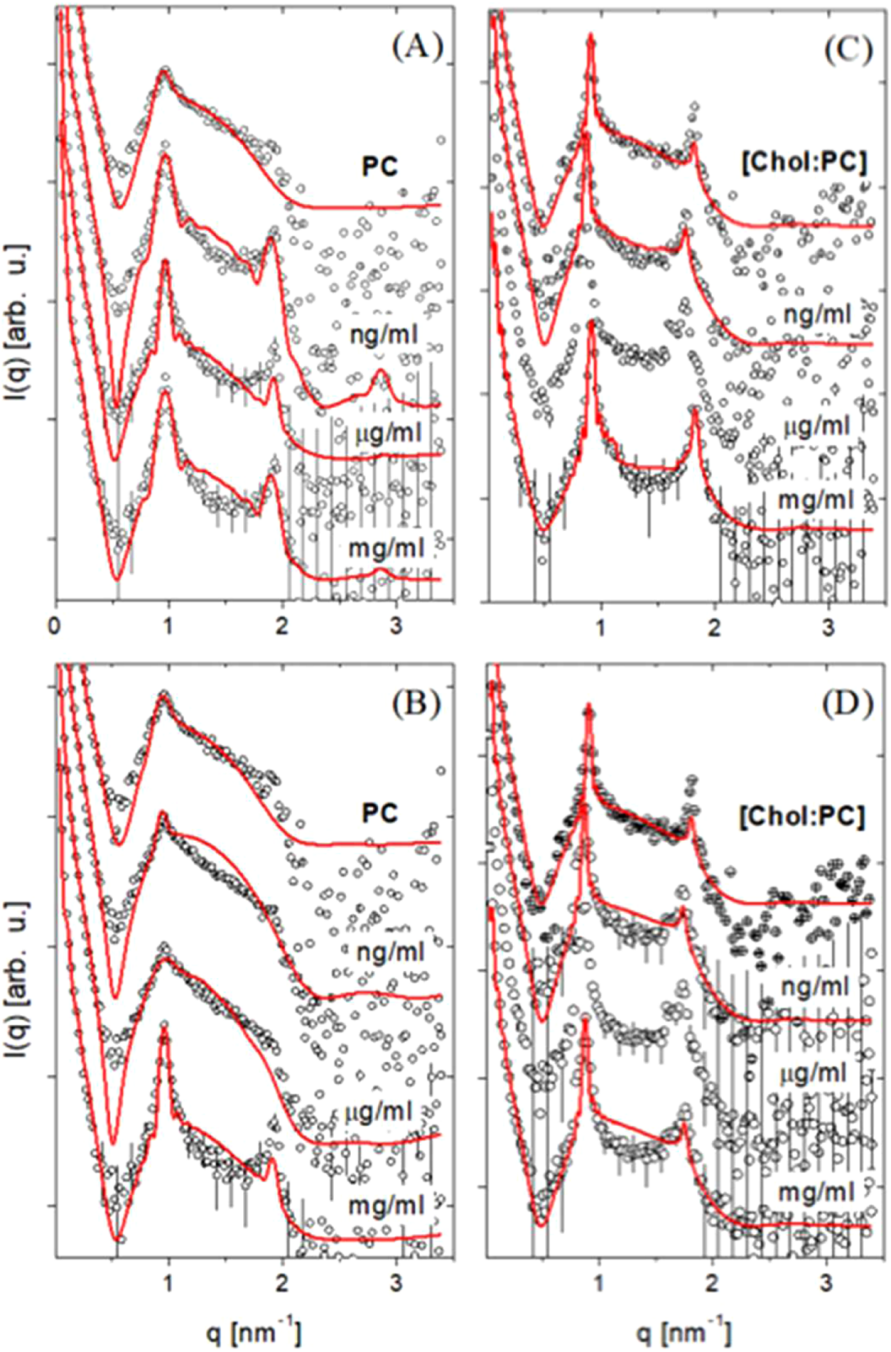
Results from small angle X-ray scattering (SAXS) experiments on model membranes made of PC, both with and without cholesterol. (A) Scattering for lipid vesicles comprising PC and Spike protein. (B) PC with RBD. (C) Combination of [Chol/PC] with Spike. (D) Combination of [Chol/PC] with RBD. The solid lines overlaying the data points represent fits using the Gaussian model.

Figure 1 depicts the scattering intensity (*I*(*q*)) as a function of the *q* vector for PC vesicles in the presence of Spike (Figure 1A,B) and RBD (Figure 1C,D). Across all SAXS data, we observed Bragg’s peaks with a 1:2:3 ratio, indicative of multilamellar vesicles. Below these scattering peaks, a pronounced bump scattering is evident, suggesting a significant presence of unilamellar vesicles in the solution.

Comparing the SAXS curves of PC vesicles with and without cholesterol (Figure 1C,D) shows a noticeable increase in peak intensity. A similar trend is evident when proteins are introduced to the PC samples. For Spike, these changes manifest at concentrations as low as ng mL^–1^, while for RBD, the effect is perceptible only at mg mL^–1^ concentrations. This suggests that the protein presence enhances membrane order by increasing the number of stacked bilayers or reducing membrane flexibility.

These preliminary observations provide a qualitative understanding of the lamellar system. Further, we undertook a global fitting of the SAXS data (Figure S3),^33^ which quantified shifts in structural and flexibility parameters such as lamellar periodicity, number of correlated layers, electron density profile, and the Caillé parameter.^41,42^ In Figure 1, the red lines represent the final fitting for each data set, showcasing a commendable fit across the entire *q* range of experimental data.

Figure 2 displays structural and elasticity parameters derived from fitting the experimental curves, plotted as protein concentration functions. The lamellar periodicity (*D*) for the unadulterated membrane systems (PC and [Chol/PC]) aligns well with values documented in the literature (Figure 2A).^43^ When analyzing the behavior of the D parameter in the presence of protein, membranes constituted solely of PC lipids exhibit no discernible changes. Contrastingly, membranes containing cholesterol display notable deviations. Specifically, an initial rise in the *D* parameter is observed for the [Chol/PC] membrane. As more protein is introduced to the solution, the lamellar periodicity of the lipid membrane begins to decrease. This effect is markedly pronounced with the Spike protein, where the difference in value (Δ*D*) reaches 0.4 nm. Such findings suggest that the protein may interface with the lipid membrane, augmenting interbilayer interactions.

**Figure 2:**
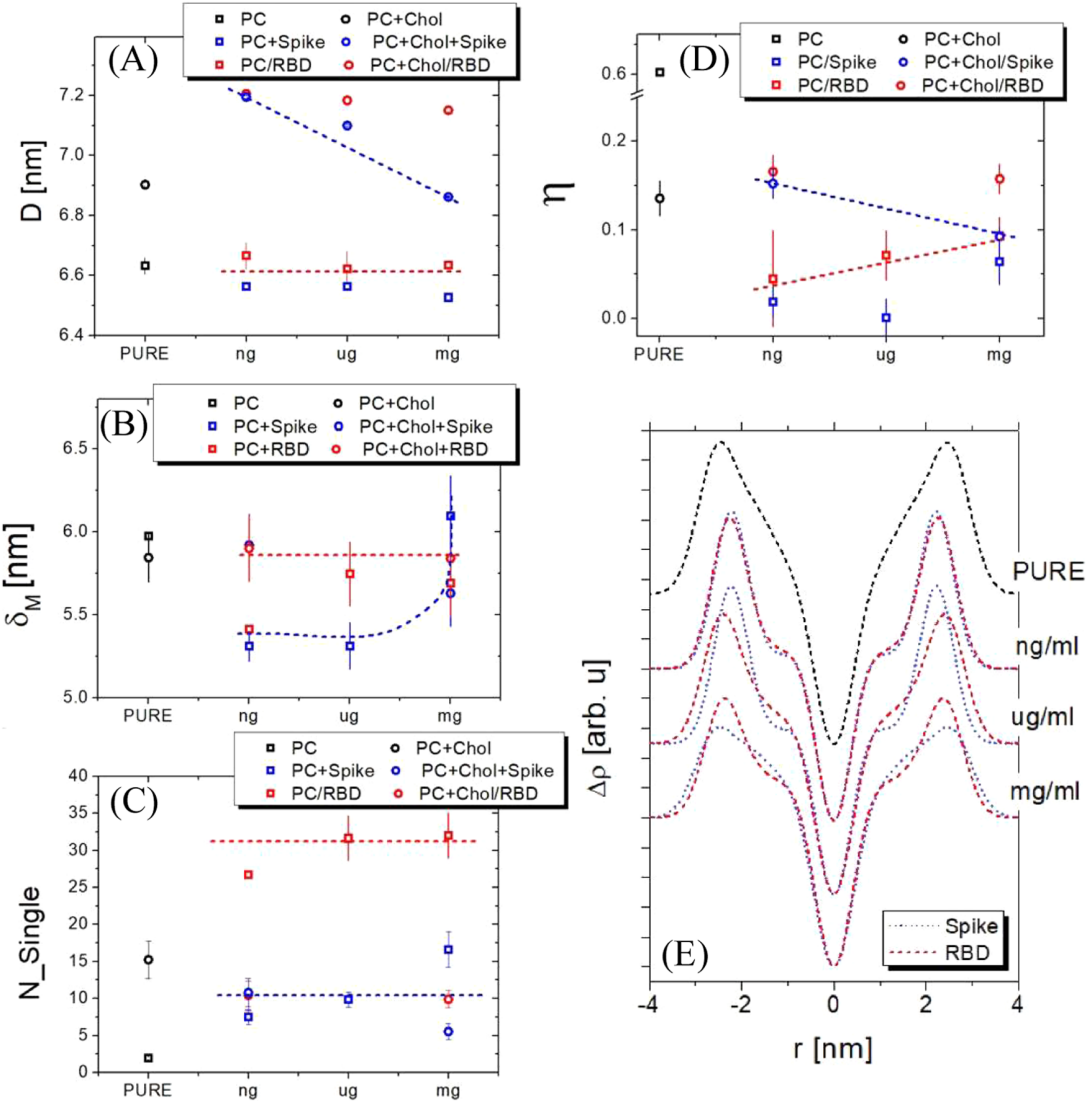
Structural and elastic parameters of vesicles with a [Chol/PC] ratio of 0.25 were examined across varying protein concentrations ranging from ng mL^–1^ to mg mL^–1^. (A) Lamellar periodicity (*D*); (B) Membrane thickness (δ_M);_ (C) Number of unilamellar vesicles *N*_single_; (D) Caillé parameter (η) and (E) Electron density profile (Δρ). Blue and red lines are provided to guide the eye in tracking the behavior of the parameters.

The membrane thickness (δ_M_) was determined as previously described^44^ and is presented in Figure 2B. The δ_M_ values for the pure membranes (PC and [Chol/PC]) align with results reported in the literature.^45^ We observed distinct behaviors for δ_M_ depending on the protein system. Both membrane compositions maintained a consistent membrane thickness value of approximately 5.9 nm for RBD. In contrast, with increasing concentrations of the Spike protein, the δ_M_ value rose to around 6.2 nm. This increase in membrane thickness further suggests the presence of the protein within the membrane, as indicated in Figure 2A.^46^

The number of stacked bilayers (*N*_bilayer_) for each membrane composition is displayed in Figure 2C. For the PC membranes in the presence of proteins, an increase in *N*_bilayer_ is observed, confirming our earlier suggestion. However, for the [Chol/PC] membrane, this value remains consistent. Another insight from the SAXS data relates to the contribution of unilamellar vesicles (ULV), represented by (*N*_single_).

In Figure 2C, a significant variation in *N*_single_ is apparent when cholesterol is present in the membrane. Introducing cholesterol to the membrane causes the amount of *N*_single_ to decrease by about 3-fold. This suggests that the combined presence of cholesterol and protein promotes greater vesicle bonding, leading to MLV formation.

In Figure 2D, the Caillé parameter (η) is plotted as a function of the protein concentration. Notably, when cholesterol is inserted into the membrane, the η value declines, signaling the formation of more rigid membranes. Such behavior aligns with expectations; incorporating up to 30% cholesterol in a molar ratio within PC membranes can induce the formation of a local gel phase. Given that this phase is less flexible than fluid phases,^4^ the observed rigidity is anticipated. While the term “rafts” conventionally refers to microdomains enriched with sphingolipids and proteins, in our model system, cholesterol modulates the order and packing density of lipid membranes, forming rigid domains that share functional similarities with raft-like behavior in facilitating protein interactions. These rigid domains highlight cholesterol’s broader role in enhancing membrane organization and stability, which may be critical for the interaction and embedding of viral proteins, especially in cholesterol-rich regions.

Adding proteins to lipid membranes changes the η parameter across both membrane compositions. An increase in the η parameter is observed for PC membranes, implying that the Spike protein and RBD may promote the formation of more flexible bilayers. Conversely, the Caillé parameter diminishes in cholesterol membranes, indicating a more rigid membrane. It seems that Spike and RBD tend to integrate within cholesterol-rich microdomains, potentially leading to the formation of defects in the membrane.

In Figure 2E, we compared the electronic contrast density (Δρ) of the PC membrane with systems containing proteins. The positive region of the Δρ corresponds to the polar region of the membrane, while the negative region represents the carbon chain.^47^ The Δρ for PC obtained in our study are in accordance with findings reported in the literature.^33^ Upon introducing proteins, subtle alterations in the Δρ are noticeable. With the Spike protein, changes appear in the negative region, accompanied by increased membrane thickness. In comparison, variations are confined to the polar region for RBD. These observations suggest that the Spike protein may penetrate deeper into the membrane while RBD likely remains on the membrane surface.

Our SAXS findings imply that SARS-CoV-2 proteins may directly interact with lipid membranes, bypassing the need for the angiotensin-converting enzyme 2 (ACE2) receptor. This agrees with previous literature reports,^20^ which conjecture the S1 protein region can associate with neutral membranes, supporting our observations. However, the SAXS data presented here indicates that the nature of the interaction between lipid membranes and these proteins varies, leading to distinct protein attachments within the bilayers.

### Langmuir Trough Experiments

To delve deeper into the events on the membrane surface, we conducted monolayer studies using the Langmuir technique, as illustrated in Figure 3.

**Figure 3:**
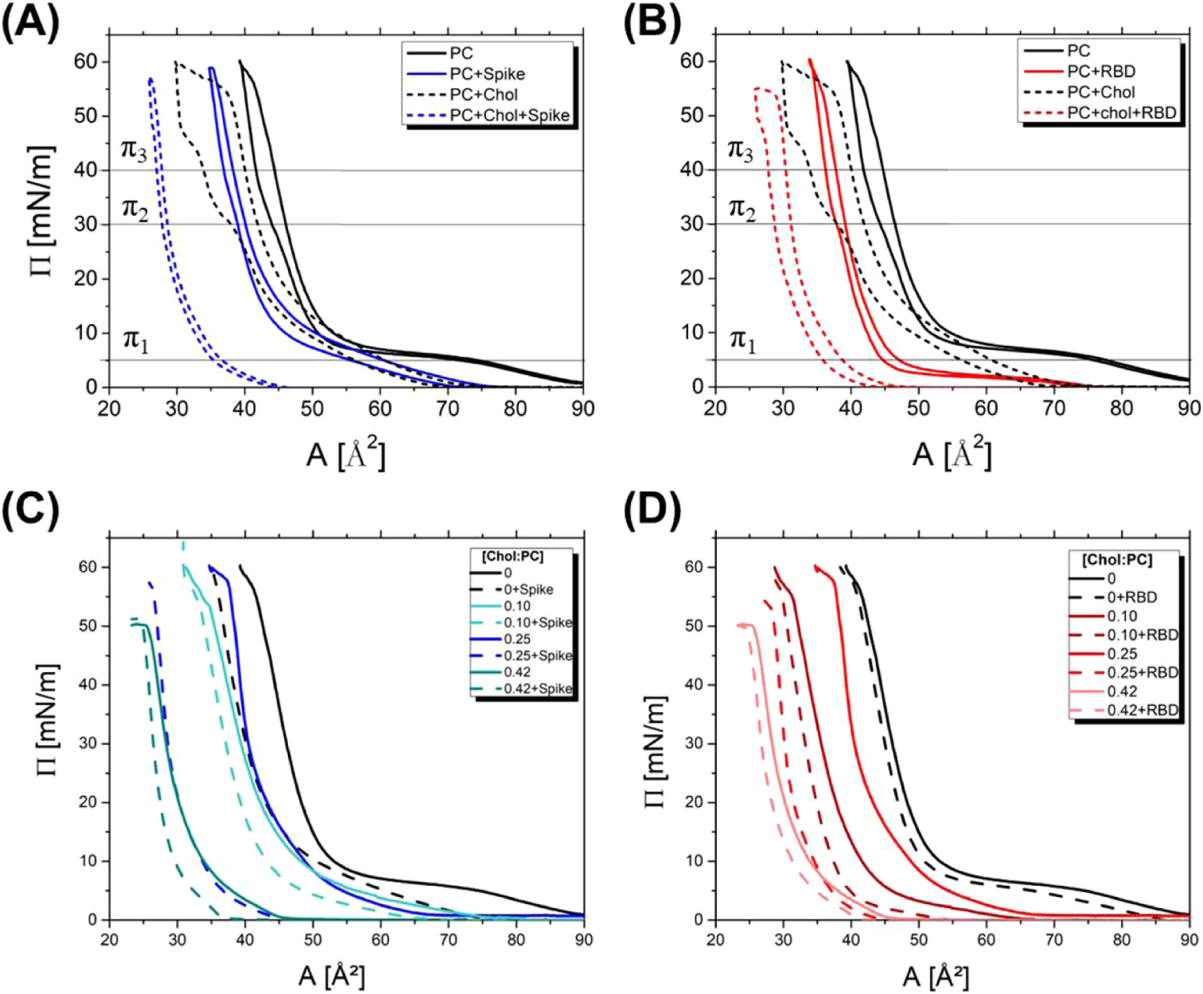
Surface pressure isotherms plotted against the molecular area. A solid black line represents PC alone; a dotted black line indicates varying [Chol/PC] ratios. In (A) and (B), the blue lines show the effect of adding Spike protein, and the red lines indicate the influence of RBD, respectively. In (C) and (D), isotherms of surface pressure per average molecular area are shown for different [Chol/PC] ratios, namely 0.10, 0.25, and 0.42. (C) Blue dashed lines represent Spike protein at a 0.1 mg/mL concentration peak, while (D) red dashed lines represent RBD at the same concentration.

Figure 3A,B depict the isotherm adsorption as a function of the mean molecular area in the absence and presence of Spike and RBD, respectively. For the PC monolayer, the behavior is in line with descriptions in the literature,^48–50^ where the transition from liquid-expanded (LE) to liquid-condensed (LC) phases occurs around 50 mN m^–1^, and monolayer collapse is observed near 65 mN m^–1^. When cholesterol is introduced into the membrane (as seen in Figure S4), there is a shift toward a smaller molecular area. As cholesterol concentration increases, this value continues to decrease. Specifically, a cholesterol range of 10% to 30% in PC membranes fosters the formation of gel phases (*L*_β_) domains. This modifies the organization of lipid molecules, resulting in regions that exhibit increased order.^4^

Figure 3 shows the monolayer composed of [Chol/PC] = 0.25, the same one studied in the SAXS experiments. When proteins are added to the water subphase, a distinct behavior emerges between the PC membrane with and without cholesterol. For the pure PC membrane, a reduction in the molecular area (Δ*A* = *A*_membrane_ – *A*_membrane+protein_) is evident, with Δ*A* values of approximately 15 Å^2^ for Spike and no change for RBD. Yet, upon the inclusion of cholesterol in the membrane, a decrease in the molecular area is observed for both monolayer compositions, yielding Δ*A* values of about 30 Å^2^ for Spike and 15 Å^2^ for RBD. Such shifts can be attributed to protein incorporation into the monolayer. This leads to a more compact lipid system locally, causing a reduction in interactions among the electric dipoles in the polar heads.^4,51^ The large area variation in the presence of the Spike protein suggests it induces more outstanding membrane alterations than RBD. Moreover, cholesterol appears to significantly influence the integration of these proteins into the lipid monolayer.

We conducted similar experiments for various [Chol/PC] ratios (as shown in Figure S5). In these experiments, we observed a decrease in the molecular area in the presence of both Spike and RBD. Interestingly, this reduction becomes steeper as more cholesterol is added, endorsing the notion that cholesterol facilitates protein insertion into the membrane. Again, it is noteworthy that the presence of Spike induces more substantial alterations in the membrane compared to RBD, as evidenced by the significant variations in the molecular area caused by lateral pressure.

To draw parallels with the SAXS experiments, we escalated the protein amount in the water subphase, ranging from ng mL^–1^ to μg mL^–1^. Using a concentration of mg mL^–1^ is not feasible since the high initial protein concentration prompts precipitation. For both protein concentrations of 0.5 and 1 mg mL^–1^, minor alterations in the monolayer are seen compared to a concentration of 0.1 mg mL^–1^. These alterations manifest as a notable decrease in the average molecular area. This observation shows that increasing protein concentration does not necessarily enhance protein incorporation into the membrane. Instead, it may promote protein aggregation at the membrane interface.

The elastic behavior of the monolayers can be assessed using the compressibility modulus (C_S_^–1^) parameter (as defined in eq 1). Standard values were observed for the pure PC system. However, when cholesterol is introduced into the monolayer composition, this value increases significantly,^44^ leading to a more rigid membrane (Figure S6).^52^ When proteins are added, no obvious change in the C_S_^–1^ is detected for the PC-only membrane. Nevertheless, there is a slight reduction in the C_S_^–1^ in the presence of cholesterol. This effect becomes more accentuated in the system containing the Spike protein (as depicted in Figure S6A,B). Similar trends persist as the [Chol/PC] ratio in the monolayers increases. These findings hint that the protein likely embeds itself in regions enriched with cholesterol, causing localized disruptions in the membrane and resulting in a more flexible monolayer

1 $$C_{S}^{-1}=-A{\left(\frac{\delta \pi }{\delta A}\right)}_{T}$$

Another widely used technique for characterizing Langmuir films is surface potential (SP).^53,54^ The SP values we obtained for the PC membranes align well with those in the literature.^55^ When cholesterol is introduced into the monolayer, a decrease in surface potential takes place, suggesting that as cholesterol content increases, the dipole interaction among the lipid heads is reduced.^56^

To discern the impact of the proteins on the SP, we used the variation value of the SP (ΔSP), defined as ΔSP = SP_WP_ – SP_P_. Here, SP_WP_ represents the surface potential of the lipid monolayer without protein, while SP_P_ denotes the monolayer with the protein. A slight reduction in the SP value was observed for the pure PC membrane with either protein, suggesting minimal protein interaction with the lipid membrane’s polar region. Interestingly, in the [Chol/PC] membranes with the Spike protein, we noticed a significant reduction in SP, roughly ΔSP = 200 mV. This further implies that cholesterol enhances protein insertion into the monolayer, thus altering the membrane’s surface charge. Conversely, RBD only achieved half the ΔSP value of the Spike protein, suggesting that it might not penetrate as effectively in the membrane interface.

Table 1 provides Δ*SP* values for various [Chol/PC] ratios. Notably, as the cholesterol content increases, the ΔSP value in the presence of Spike also rises, indicating heightened protein incorporation into the monolayer. For RBD, however, the ΔSP remains consistent across all [Chol/PC] ratios. This consistency suggests that, despite increasing cholesterol content, the extent of RBD integration in the membrane remains unchanged.

**Table 1:** ΔSP Values for the PC Monolayer with and without Cholesterol in the Presence of Both Spike and RBD

[Chol/PC]	ΔSP_Spike_ (mV)	ΔSP_RBD_ (mV)
0	0	0
0.10	50	100
0.25	200	100
0.42	400	100

### Atomic Force Microscopy

We conducted AFM measurements to examine the monolayer topography in the presence and absence of cholesterol and proteins (Figure 4), transferring the monolayers onto mica surfaces via the Langmuir–Blodgett (LB) technique. Figure 4A,B depict the AFM images of monolayers composed of PC alone and a combination of [Chol/PC] = 0.25 molar ratio. In the PC AFM images (Figure 4A), we observed cavities approximately 20 nm in diameter.^57^ In contrast, the [Chol:PC] images (Figure 4B) displayed a denser lipid monolayer with fewer and smaller cavities, around ∼ 16.5 nm in size. This suggests that the monolayer containing both PC and cholesterol is more compact than that of PC alone, corroborating our findings from the SAXS experiments, adsorption isotherms, and compressibility modulus values. For the monolayers containing Spike (Figure 4C,D), conspicuous regions with heights of roughly 9.5 nm are evident, indicating protein clusters integrated within the lipid monolayer. Notably, these clusters are more abundant in monolayers that include cholesterol, further validating our earlier data. In the case of RBD (Figure 4E), smaller clusters are visible, yet they are less prominent than in the Spike protein-containing system. Interestingly, in Figure 4F, cholesterol in the monolayer did not lead to any detectable height variation compared to Figure 4B.

**Figure 4:**
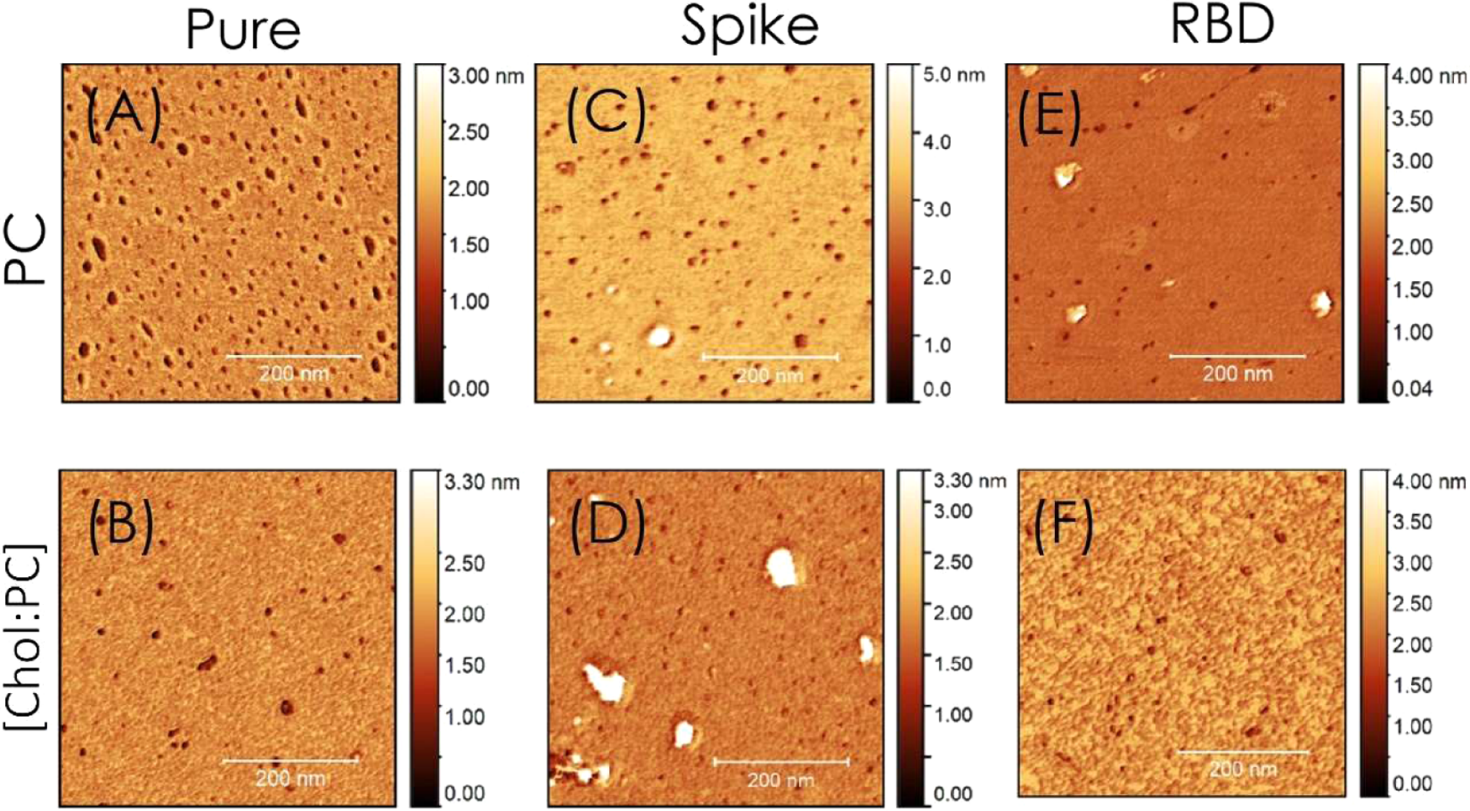
AFM images illustrate the influence of cholesterol on the lipid monolayer. (A) Pure PC monolayer, (B) [Chol/PC] = 0.25 monolayer, (C) Pure PC monolayer with Spike protein, (D) [Chol/PC] = 0.25 monolayer with Spike protein, (E) Pure PC monolayer with RBD, (F) [Chol/PC] = 0.25 monolayer with RBD.

### Electrochemical Characterization

Following the confirmation of protein insertion into the monolayers and vesicles, we verified the bioavailability of antigenic sites through electrochemical experiments. These aimed to assess the biorecognition capacity of the proteins inserted in the monolayer when interacting with monoclonal antibodies against the RBD domain of the Spike protein (Anti-RBD). To achieve this, we transferred the monolayers via Langmuir–Schaefer (LS) onto glass surfaces coated with tin-doped indium oxide (ITO) thin films and conducted electrochemical tests, such as cyclic voltammetry (CV) and electrochemical impedance spectroscopy (EIS).

Spike and RBD have been utilized as biorecognition elements in developing biosensors.^58–60^ Castro et al. used electrochemical tests to analyze both proteins in the presence of gold nanoparticles (AuNPs). They demonstrated a strong dependence on the bioavailability of the proteins for effective interaction with immunoglobulin-like antibodies.^61^ These interactions rely on the specificity of the antibody’s combinatorial site, which binds the antigen with high affinity, represented by a close complementarity between the three-dimensional structures of the two molecules.^62^ Such interactions can be evaluated electrochemically by indirect detection, where the analytical response is derived from a redox probe dissolved in solution.

CV is an electrochemical technique widely used for evaluating oxidation–reduction processes and characterizing materials owing to its easy execution and rapid analysis.^63,64^ The anodic and cathodic peak currents versus the square root of the sweep rate, obtained from the clean ITO electrode and modified with [Chol/PC] = 0.30 monolayer in the presence of Spike and RBD at concentrations of 0.1 and 1 mg mL^–1^, exhibited linearity in the cathodic and anodic peak currents (Figure S7). This linearity indicates thermodynamically favorable electron transfer with an expected electrochemical signal pattern, which is diffusion-controlled. As the sweep rate increases, the oxidation/reduction peak current also gradually increases, inducing a limitation in charge transfer kinetics, characteristic of a semireversible system. We hypothesize that the platform constructed using ITO/[Chol/PC] + biomolecules present some resistive path for electron flow from the electrolyte to the substrate, leading to a decrease in the oxidation intensity of the spikes.

Given that these are semireversible and diffusion-controlled systems, we can apply the Randles-Sevcik equation (eqs S6 and S7) to determine the electroactive area of the modified electrodes, as shown in Table 2.

**Table 2:** Electroactive Area of ITO Modified with the [Chol/PC] = 0.30 Monolayer at Different Concentrations (0.1 and 1 mg mL^–1^) of Spike and RBD

composition	electroactive area (10^–2^ cm^2^)
0 (in ITO)	2.0
[Chol/PC] + Spike-0.1	0.18
[Chol/PC] + Spike-1	1.1
[Chol/PC] + RBD-0.1	0.82
[Chol/PC] + RBD-1	1.3

From the data in Table 2, we can infer that the monolayers containing the proteins were successfully collected and directly influenced the electroactive area. This indicates good adhesion in the ITO since all membranes had signs of decay in the area compared to the clean ITO (0.02 cm^2^). This is due to hydrocarbon chains derived from lipids, which are insulating structures and thus impair electron transfer.

In addition, it is certain that the concentration of RBD and Spike, which are charged structures, also directly affects the values of the electroactive area because the latter increases as protein concentration rises.

In the analysis of voltammograms (Figure 5A,C), we detected that the monolayers modified with Spike-0.1 decreased the intensity of the peaks by approximately 13%. At the same time, in Spike-1, it was 42%, indicating that the biomolecule concentration reflected in the electron transfer that occurs at the electrode–solution interface. Based on the lower peak intensity in the voltammograms and the increase in the semicircle observed in the Nyquist diagrams (Figure 5B,D), the formation of the antigen–antibody complex in the presence of anti-RBD antibodies and the consequent retention of the latter on the surface would occur only using Spike-0.1 concentration. With Spike-1, the values are almost unchanged, which suggests a possible aggregation, compromising the bioavailability of the active sites and preventing the biorecognition of anti-RBD IgG. These data underline the importance of the protein concentration used to construct the [Chol/PC] + Spike-0.1 monolayer in easing the antigen–antibody interaction.

**Figure 5:**
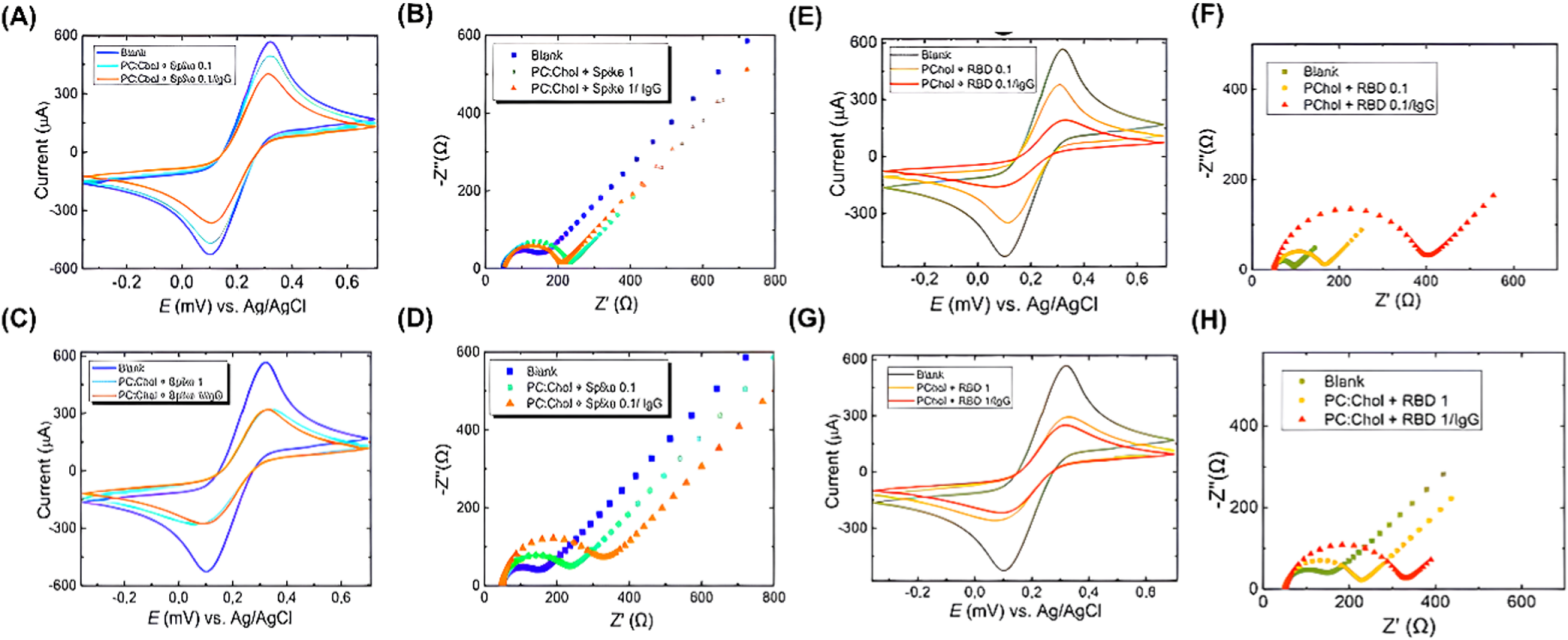
Electrochemical study of ITO modified with [Chol/PC] + Spike monolayers in the presence and absence of anti-RBD antibodies. (A) Cyclic voltammogram and (B) Nyquist diagram of the [Chol/PC] + Spike-0.1 modification. (C) Cyclic voltammogram and (D) Nyquist diagram of the [Chol/PC] + Spike-1 modification. Electrolyte: K4Fe­(CN)­6/K3Fe­(CN)­6 5 mmol L^–1^ solution in KCl 0.1 mol L^–1^. ITO modified with [Chol/PC] + RBD monolayers in the presence and absence of anti-RBD antibodies. (E) Cyclic voltammogram and (F) Nyquist diagram of the [Chol/PC] + RBD-0.1 modification. (G) Cyclic voltammogram and (H) Nyquist diagram of the [Chol/PC] + RBD-1 modification. Electrolyte: K_4_Fe­(CN)_6_/K_3_Fe­(CN)_6_ 5 mmol L^–1^ solution in KCl 0.1 mol L^–1^.

For RBD-0.1 (Figure 5E,G), we can observe a reduction in the intensity of the peaks of approximately 34%, while for RBD-1, it was 42%. In Figure 5E, we can see a decrease in the intensity of the peak of 48% in the presence of anti-RBD antibodies. In contrast, we did not observe this decay in Figure 5G. Therefore, we believe that systems that use the most concentrated RBD may present an unfavorable conformation on the surface, resulting in the reduction or even exclusion of active sites for recognition.

The EIS tests (Figure 5F,H) described similar phenomena where the lowest concentration of RBD promoted the most significant increase in *R*_ct_ values in the presence of anti-RBD, a sign of antigen–antibody interaction. The results suggest that the interaction points can be increased by decreasing the number of active sites on the electrode surface. That is, there is a minimum concentration that can generate an electrochemical signal while maintaining the target, achieving better sensitivity for constructing a biosensor.^65,66^ Concentrated proteins can form aggregates on the surface, exhibiting an unfavorable conformation and decreasing or even excluding active sites for recognition.

A calibration curve was performed with the Spike protein (Figure S8A) and RBD (Figure S8B) to understand active site distribution better, obtaining detection limit values of 364.1 and 205.2 ng mL^–1^, respectively. Thus, it is reasonable to propose the Langmuir monolayer collection method for possible applications as an electrochemical biosensor. Furthermore, the use of RBD would be preferred over Spike as the former macromolecule made an improvement in the detection limit possible. This was, in fact, an expected outcome, bearing in mind previous reports in the literature about the benefits of diminishing the receptor size of the target, thereby reducing the electronic steric effects that the other regions of the molecule can promote. In turn, this would make biorecognition and response easier, bringing about an improvement in detection sensitivity.^61,66,67^

The platform was tested without protein (Figure S9), and results indicated that the antibodies do not interact with the lipid monolayer. This finding rules out the possibility that the monolayer affected the specificity and efficacy of the antibody-ligand interaction. Moreover, it underscores the crucial role of proteins as biorecognition agents, facilitating more effective therapeutic strategies.

Additionally, tests conducted without cholesterol (Figure S10) revealed that the structural organization necessary for anti-RBD binding occurs only in its presence. This highlights cholesterol’s role in promoting lipid raft formation, critical in organizing the monolayer for optimal antibody binding. These insights emphasize cholesterol’s function in modulating membrane organization, which may influence the design of future therapeutic platforms.

### Molecular Dynamics Simulations

Figure S11 presents the Root Mean Square Deviations (RMSD) for the models comprising the lipid monolayers in complex with RBD and Spike. Visual inspection of the protein RMSD time evolution reveals that all systems reached equilibrium (after the 100 ns mark) and maintained conformational stability throughout the simulations. Graphs for RBD-monolayer (Figure S11A) and Spike-monolayer (Figure S11B) complexes present similar fluctuation patterns over time for the three different [Chol:PC] ratios, namely 0, 0.1, and 0.3, with average values ranging from 1.5 to 4 Å approximately for the RBD systems and from around 4 to 6 Å for the Spike ones. Accordingly, cholesterol concentration does not significantly influence the systems’ dynamical properties.

To evaluate the rigidity of the lipid monolayers, the parameter known as area per lipid (APL) was computed through the whole MD trajectories. The APL gets reduced as cholesterol concentration increases for the bare monolayers, thereby decreasing the monolayer’s total area. The average percentage decrease in APL between the 0 and 0.10 molar ratio cholesterol models is almost 5%. An additional 10% average decrease is observed for the [Chol/PC] = 0.30 monolayer (check results in Table 3). Hence, the overall APL average percentage decrease between the pure PC monolayer and the 30% cholesterol one is over 14. The reduction in monolayer total area should be proportional to this figure. The APL reduction indicates that the lipid molecules are less accessible to move in the presence of cholesterol because of the decreased available space. This translates into a more rigid monolayer as the number of cholesterol molecules goes up. When RBD and Spike were added to the systems, APL was further reduced for the three [Chol:PC] monolayer models. For the cholesterol-free monolayer, the average APL decrease was 9% and 21% upon RBD and Spike binding, respectively (Table 3). In turn, for the [Chol/PC] = 0.10 monolayer, the average descent was 14% and 23% after RBD and Spike binding, respectively, and 8% and 17%, respectively, in the [Chol/PC] = 0.30 model. Then, the monolayer total area is smaller when complexed with both RBD and Spike, with the latter producing more significant variations for the three cholesterol concentrations studied. At the same time, the monolayer thickness slightly increases for all systems as the cholesterol molar ratio goes from 0 to 0.30 of membrane composition (data not shown). Naturally, as lipids come closer to occupy less horizontal surface, the width of the membrane is augmented. These results correlate nicely with those discussed earlier for the Langmuir technique experiments.

**Table 3:** APL Values (In Å^2^ Plus Standard Deviations) of Lipid Monolayers with [Chol/PC] Molar Ratios of 0, 0.10, and 0.30, Averaged Over the Final 100 ns of the 200 ns MD Trajectories

	[Chol/PC]		
systems	0	0.10	0.30
[Chol/PC]	69.19 (0.70)	66.04 (0.94)	59.70 (1.10)
[Chol/PC] + RBD	63.10 (0.85)	57.79 (1.46)	55.17 (1.60)
[Chol/PC] + Spike	54.50 (0.39)	50.73 (0.64)	50.18 (0.81)

To measure the stability of the monolayer-protein complexes at a molecular level, the nonbonded (van der Waals and Coulomb) interaction energies for the three [Chol/PC] lipid monolayers were calculated along the MD trajectories. Results are presented in Table 4. For RBD, energy values point to a stronger nonbonded interaction with the two monolayers featuring cholesterol. The total nonbonded energy average of RBD with the pure PC monolayer (0% cholesterol) is −192.70 kcal/mol, which indicates a somewhat favorable binding. Meanwhile, the total nonbonded energy averages for the 0.10 and 0.30 molar ratios of cholesterol monolayers are −314.32 and −492.20 kcal/mol, respectively. These results reveal that the protein–membrane complexes are more stable in the presence of cholesterol, with stability increasing as cholesterol concentration is augmented. Similarly, the Spike protein’s energy values demonstrate a stronger nonbonded interaction for the [Chol/PC] membranes. In this case, the total nonbonded energy average with the membrane in the absence of cholesterol is −1244.29 kcal/mol while the total nonbonded energy averages for the 0.10 and 0.30 molar ratios of cholesterol monolayers are −1752.91 and −2703.02 kcal/mol, respectively. At variance with the RBD results, the energy gap between the 0.10 and 0.30 molar ratios is proportionally larger than the corresponding gap between the 0 and 0.10 ones. In summary, the stabilization effect of cholesterol is observed for both RBD and Spike, with the latter benefiting to a greater degree from high membrane contents of cholesterol.

**Table 4:** Electrostatic (Ele), van der Waals (vdW), and Total (Tot) Nonbonded Interaction Energies (In kcal/mol Plus Standard Deviations) between SARS-CoV-2 Proteins and Lipid Monolayers with [Chol/PC] Ratios of 0, 0.10, and 0.30, Averaged Over the Final 100 ns of the 200 ns MD Trajectories

proteins	[Chol/PC]	vdW	Ele	Tot
RBD	**0**	–62.59 (20.79)	–130.11 (68.78)	–192.70 (82.10)
	**0.10**	–96.86 (29.96)	–217.46 (91.82)	–314.32 (111.79)
	**0.30**	–123.45 (24.37)	–368.75 (92.75)	–492.20 (109.54)
spike	**0**	–272.98 (43.88)	–971.31 (193.22)	–1244.29 (207.58)
	**0.10**	–291.97 (73.44)	–1460.94 (229.65)	–1752.91 (293.44)
	**0.30**	–316.15 (38.21)	–2386.87 (194.79)	–2703.02 (205.97)

Figure S12 shows the time evolution of the contact surface areas between either RBD (Figure S12A) or Spike (Figure S12B) with the membrane at different cholesterol concentrations. For RBD, the contact surface area with the cholesterol-free monolayer rapidly diminishes from its initial value just above 3000 Å^2^ and, after system equilibration, the curve oscillates around the average value of 1625 Å^2^ for the rest of the simulation.

The initial decrease in contact surface area is also verified for the monolayer at [Chol/PC] = 0.10. This time, the graph fluctuates around slightly lower values, i.e., in the range of 900 and 1300 Å^2^, with an average value of 1227 Å^2^. Contrary to these data, the evolution of the contact surface area for the 0.30 [Chol/PC] ratio monolayer is much steadier. The curve suffers a less steep decline from its initial value in excess of 3500 Å^2^ and stabilizes around the 2500 Å^2^ mark, the average amounting to 2859 Å^2^. The results indicate that, regardless of the presence of cholesterol, the macromolecule remains attached to the membrane but, at high cholesterol contents, the contact surface area is markedly larger. As for the Spike protein complexes, it has to be said that the protein also experiences a reduction in the initial contact surface area with the lipid monolayer for all [Chol/PC] ratios. Starting at a contact surface area in the range of 15,000–17,000 Å^2^, there is a rapid decrease during the first 50 ns of the trajectories, the extent of which is more important for the 0% and 10% cholesterol monolayers, with average values of 6830 and 7180 Å^2^, respectively. The corresponding value for the 0.30 [Chol/PC] molar ratio is 10,808 Å^2^. Thus, the contact surface area between the protein and the monolayer is significantly larger at high cholesterol concentration. In short, the simulation results attest to the Spike protein establishing a wider contact surface with the membranes than RBD, which suggests again a more stable binding, and that cholesterol heavily favors this ability.

The results of the afore discussed nonbonded energy and contact surface area should in principle be compatible with membrane internalization, because higher cholesterol concentrations would permit a deeper embedding of both RBD and Spike in the monolayer. The strength of the initial binding of said macromolecules to the membrane is measured by the transfer free energy (Δ*G*_T_), which amounts to the free energy of transferring amino acid side chains from an aqueous environment to the lipid monolayer. Values of Δ*G*_T_ for Spike and RBD are negative (−6.1 and −9.1 kcal/mol, respectively), proving an initial thermodynamically favorable binding. Figure 6A,B show the degree of penetration into the membrane of RBD and Spike, respectively, throughout the MD trajectory for the three [Chol/PC] monolayer models. It can be observed that, at the starting position of RBD, the maximum penetration is about 15 Å inside the monolayer for all three models. In the absence of cholesterol, RBD acquires a more superficial location in the membrane, at an average maximum penetration of some 9 Å from 100 ns onward. This result is concordant with the outcome of the contact surface area calculations. However, when cholesterol is present, this behavior is less drastic. In effect, even though the protein does not remain at the initial depth inside the membrane either, it is still considerably embedded into it. The average maximal depth attained by RBD over the last 100 ns of simulation is around 11 Å for the 0.10 molar ratio cholesterol membrane and close to 12 Å for the 0.30 one. These results are a sign that cholesterol somewhat stabilizes the interaction of RBD with the lipid membrane. On the other hand, the starting location of the protein for the Spike models corresponds to a maximum penetration of 25 Å inside the monolayer. For the noncholesterol monolayer, the position of the protein gets substantially more superficial as the trajectory progresses. However, it remains embedded inside the membrane, with a final average maximal penetration of some 12 Å. The picture is fairly different for the 0.10 and 0.30 cholesterol membranes as the penetration of the Spike protein stabilizes at an average maximal depth inside the membrane of 15 and 17 Å, respectively. Recall here that the capability of the Spike protein to embed into the lipid membranes is not mediated by its transmembrane domain since, as stated in the Materials and Methods section, the structure used in this work (both in the experimental assays and the theoretical calculations) lacks such domain. Altogether, these data strongly point to a major stabilizing effect of cholesterol on the embedding and interaction of the Spike protein with the lipid monolayer (and to a lesser degree for RBD), consistent with the outcomes of the nonbonded interaction energies, contact surface areas, and Langmuir trough experiments presented above.

**Figure 6:**
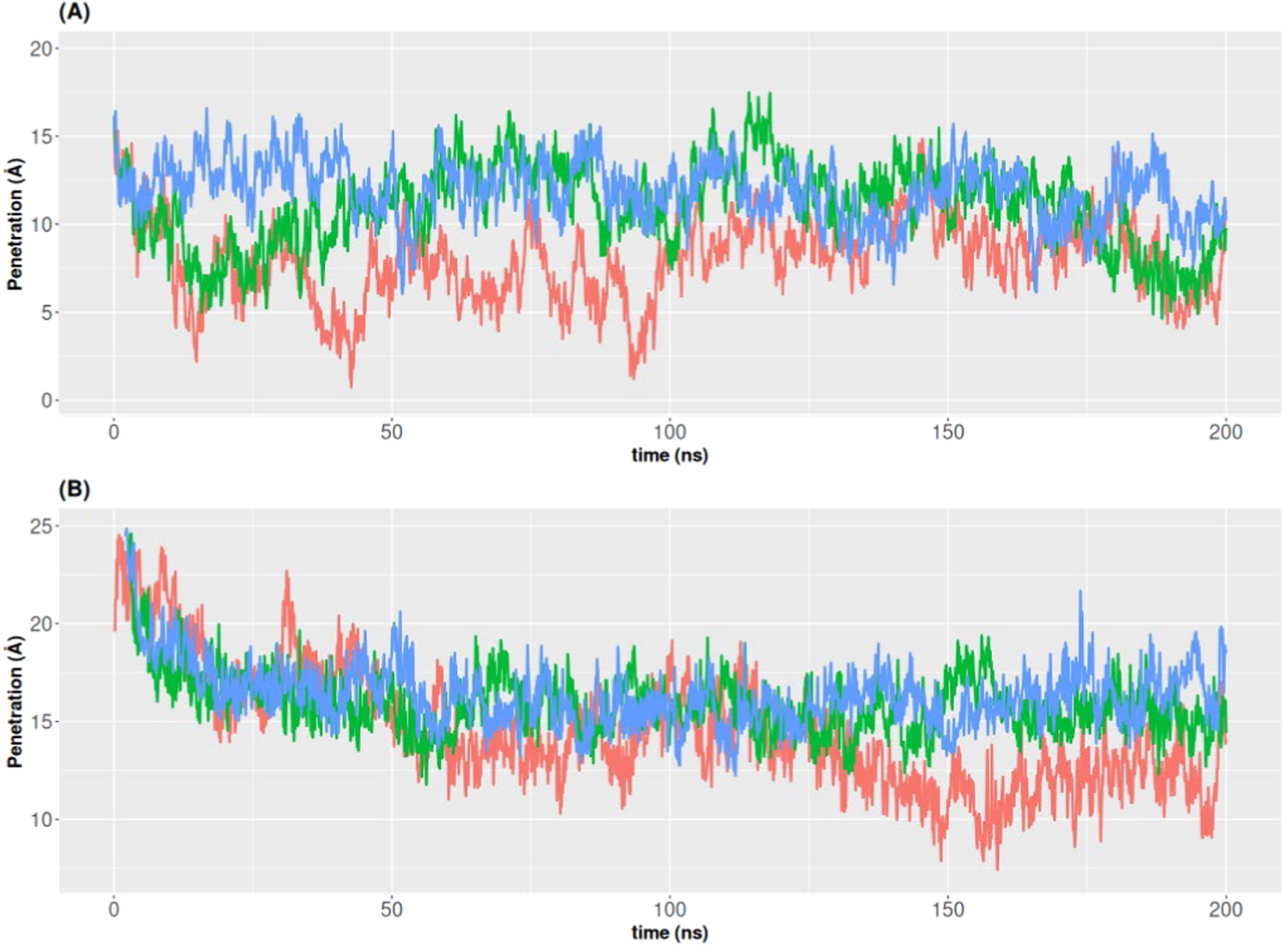
Time evolution of SARS-CoV-2 RBD (A) and SARS-CoV-2 Spike (B) membrane penetration (in Å) over the MD trajectories for the lipid monolayers at [Chol:PC] ratios of 0 (red graph), 0.10 (green graph) and 0.30 (blue graph).

As a final step in analyzing the MD simulations output, we sought to rationalize from a molecular standpoint the results derived from the electrochemical assays, particularly the differential biosensor sensitivities found in the experiments. To this effect, we calculated the solvent accessible surface area (SASA) of RBD, both isolated as well as integrated in the Spike protein, in complex with the [Chol:PC] monolayer at a 0.30 ratio (the same cholesterol concentration previously used in the electrochemical studies). Models can be visualized in Figure 7. In the case of the Spike, all three RBD domains (each one about a different subunit) were considered. A probe radius of 2.1 Å was selected. SASA measured for the isolated RBD was more prominent than those of the three RBD domains inside the Spike. Average values were 9737 (s.d.: 337) Å for isolated RBD and 7259 Å (s.d.: 274), 7068 (s.d.: 287) Å and 7607 (s.d.: 284) Å for the RBD domains in Spike chains A, B and C, respectively. As expected, RBD is more accessible to antibodies targeting its epitopes when separated from the Spike protein. This is not only due to a more extensive SASA but also, as postulated in the previous section, the absence of adjacent protein groups that could pose steric conflicts and hinder epitope recognition.

**Figure 7:**
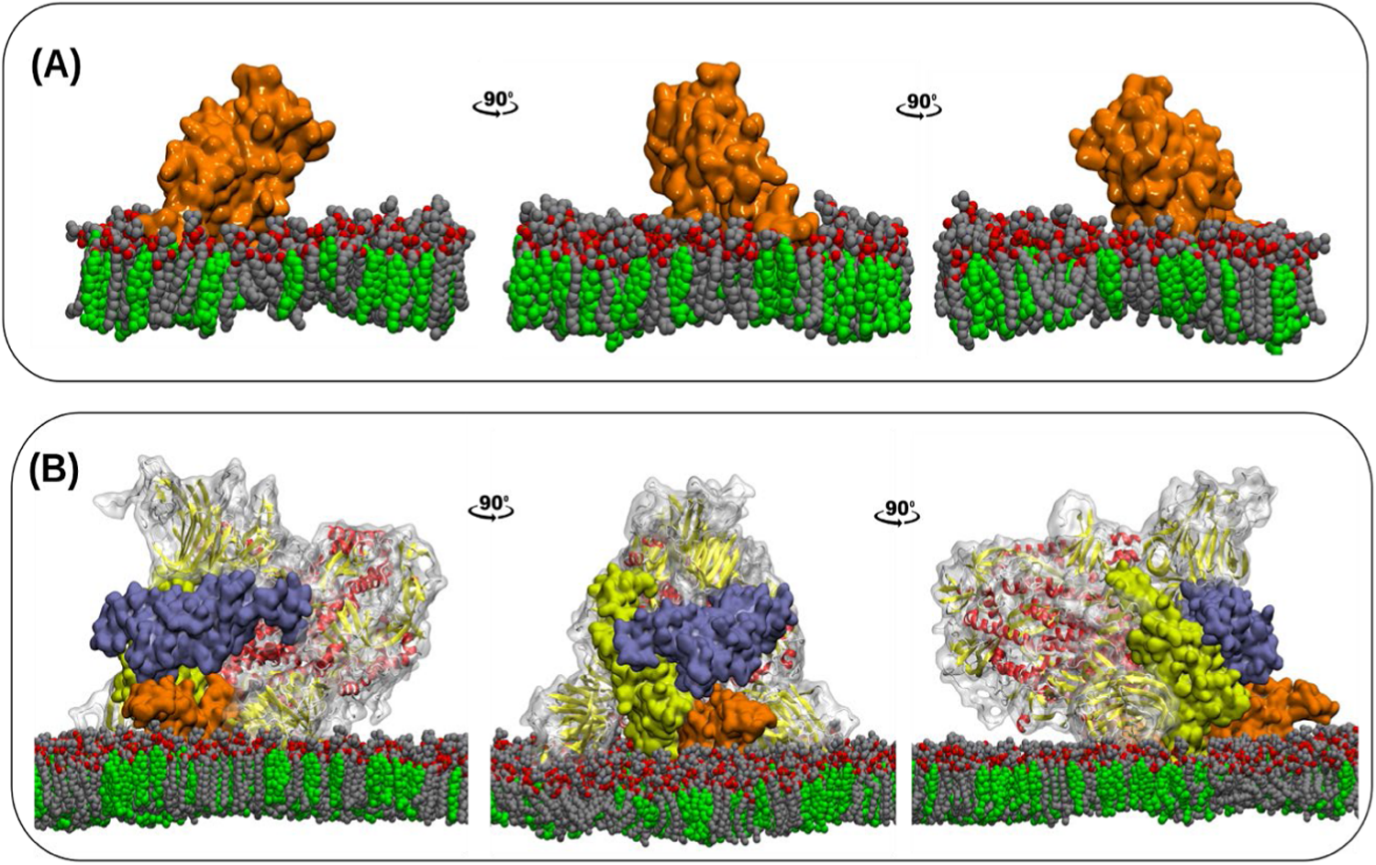
Models of SARS-CoV-2 RBD (A) and SARS-CoV-2 Spike protein (B) in complex with the [Chol/PC] = 0.30 lipid monolayer at the end of the MD simulations. Three rotated side views are portrayed for each system. PC carbon atoms are colored grey, PC oxygen atoms are colored red, and cholesterol molecules are colored green. Each RBD domain in Spike is drawn in a different color. Yellow: chain A domain, orange: chain B domain, violet: chain C domain.

This is apparent when comparing the free RBD orientations in Figure 7A with those of the corresponding domains of Spike in Figure 7B. These factors may explain why a lower detection limit, i.e., higher sensitivity, was attained with the RBD-based biosensor in the electrochemical studies.

The combined adsorption isotherm and SAXS information evidence cholesterol’s critical role in enhancing the integration of SARS-CoV-2 proteins into bilayers and monolayers. Increasing cholesterol content in the membrane significantly boosted protein insertion, indicating a preference for cholesterol-rich regions. This led to a more rigid membrane structure, characterized by the *L*_β_ phase, as confirmed by increased compressibility modulus and Caillé parameter. Electron density contrast analyses and AFM imagery suggest a strong tendency for the Spike protein to embed within the lipid membrane. At the same time, the hydrophilic regions of the RBD favor their positioning near the membrane’s polar region. Electrochemical experiments revealed that RBD-containing monolayers exhibited higher R_ct_ values when exposed to anti-RBD antibodies, implying enhanced antibody binding. These findings closely agree with those of the MD simulations, confirming the Spike protein embedding within the membrane. At the same time, the RBD is more favorably located at the surface of the membrane due to its hydrophilicity.

## Conclusions

In this study, we explored the influence of cholesterol on the structural and mechanical properties of model lipid membranes and examined how the SARS-CoV-2 Spike protein and its RBD interact with these cholesterol-enriched systems. We doped PC membranes with cholesterol in molar ratios ranging from 0.10 to 0.42, and our results confirmed that cholesterol promotes the formation of the *L*_β_ phase, as seen in the increased membrane rigidity and compressibility, corroborating previous literature reports.

We investigated the interaction of SARS-CoV-2 proteins with lipid monolayers and bilayers, and both adsorption isotherm and SAXS data demonstrated that the proteins can be associated with neutral membranes, even in the absence of the ACE2 receptor. Cholesterol-rich membranes, particularly at higher concentrations, facilitated the incorporation of the Spike protein, stabilizing its interaction with the lipid membrane. This stabilization was confirmed by increased compressibility modules and decreased electrostatic interactions, as evidenced by the Caillé parameter. However, this effect was less pronounced for the RBD, which showed a stronger affinity for the polar regions of the membrane due to its hydrophilic nature.

The electrochemical characterization revealed that protein concentration is critical in determining the bioavailability of antigenic sites and the effectiveness of antigen–antibody interactions. Lower concentrations of Spike and RBD proteins showed enhanced interaction with anti-RBD antibodies, while higher concentrations led to aggregation, limiting the accessibility of active sites. This suggests that cholesterol-rich membranes may provide a suitable environment for these interactions, which could have potential applications in biosensor development.

Molecular dynamics simulations supported experimental data, showing that higher cholesterol concentrations enhance the stability of protein–membrane interactions, mainly for the Spike protein. The simulations confirmed that cholesterol-rich regions facilitate protein embedding while the RBD remains more superficial, consistent with its hydrophilic nature. The congruence between the experimental and theoretical results further strengthens the robustness of the model we proposed in this research.

Our findings provide insights into how cholesterol modulates the interaction of SARS-CoV-2 proteins with lipid membranes, highlighting the stabilizing role of cholesterol in the membrane insertion of the Spike protein.

This study also suggests that the stabilizing role of cholesterol in facilitating the Spike protein’s interaction with lipid membranes may influence the efficiency of virus-cell fusion. This stresses the potential relevance of cholesterol-rich domains for initial viral attachment and optimizing conditions that promote membrane fusion, especially in scenarios with low ACE2 expression or availability.

These results contribute to a better understanding of the biochemical interactions between viral proteins and host cell membranes but do not yet provide direct evidence for therapeutic strategies targeting these membrane domains. Nonetheless, if the outcomes of this work hold at physiological conditions, a cholesterol-rich membrane would be more susceptible to virus infection.

## Supplementary Material



## Notes

SDS-PAGE gel showing the profile of SARS-CoV-2 Spike and RBD proteins under denaturing conditions (Figure S1); initial configuration of the simulated periodic boxes oriented along the lipid monolayer *x*–*y* axis and projected on the *z*-axis (Figure S2); examples of the SAXS data contributions (Figure S3); surface pressure isotherm as a function of molecular area at different [Chol:PC] ratios (Figure S4); surface pressure as a function of molecular area in the presence of cholesterol and increasing concentrations of Spike and RBD proteins (Figure S5); compressibility modulus associated with the incorporation of Spike and RBD into cholesterol-containing monolayers (Figure S6); electrochemical evaluation of ITO electrode surface modification with cholesterol-containing monolayers and increasing protein concentrations (Figure S7); calibration curve for Spike and RBD detection (Figure S8); electrochemical evaluation of ITO modification in the presence of antiRBD antibodies (Figure S9); electrochemical response of cholesterol-containing monolayers interacting with Spike or RBD in the presence of antiRBD antibody (Figure S10); root mean square deviations (RMSD) of [Chol:PC] lipid monolayers complexed with viral proteins over the MD simulations (Figure S11); and time evolution of the contact surface area between SARS-CoV-2 proteins and the lipid monolayers (Figure S12) (PDF)

## §

Diamond Light Source, Didcot, Oxfordshire, England OX11 0DE, United Kingdom
