# Supplementary material for: Influence of Cholesterol on the Insertion and Interaction of SARS-CoV‑2 Proteins with Lipid Membranes
Source: ACS Appl Bio Mater. 2025 Jun 6;8(6):5380–94. doi: 10.1021/acsabm.5c00776 (PMC12175161; doi:10.1021/acsabm.5c00776)
Supplement: Supplementary file 1 [file mt5c00776_si_001.pdf]

Supporting Information

Influence of Cholesterol on the Insertion and Interaction of SARS-CoV-2 Proteins with Lipid Membranes

Priscila S. Ferreira,[1#] Barbara B. Gerbelli,[1§#] Jorge Cantero,[2] Federico Iribarne,[2,*] Ana C. H. de Castro-Kochi,[1] Leandro T. Kochi,[1] Fabiola L. Castro,[1] Wendel A. Alves.[1,*]

[1]Center for Natural and Human Sciences, Federal University of ABC, Santo André, 09210-580, Brazil.

[2]Theoretical Chemical Physics and Biology Group, Mathematics-DETEMA Department, Faculty of Chemistry, UdelaR, General Flores 2124, Montevideo 11800, Uruguay.

[§]*Present address: Diamond Light Source, Didcot, Oxfordshire, England, United Kingdom*

[#] *Co-contribution*

**Corresponding author:*

Wendel A. Alves (Federal University of ABC): wendel.alves@ufabc.edu.br

Federico Iribarne (University of the Republic): fede@fq.edu.uy

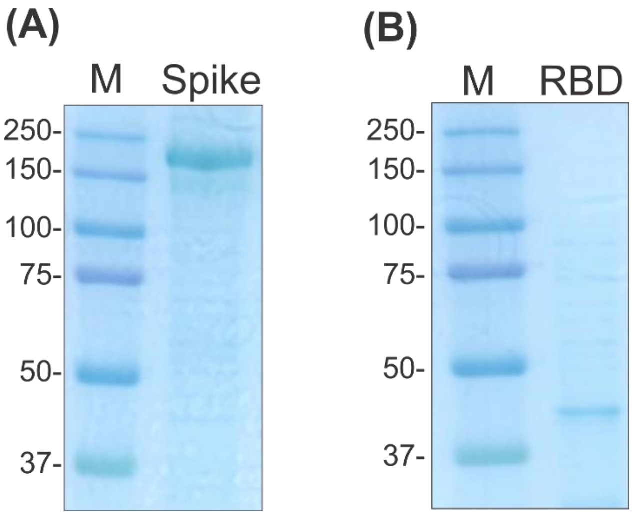

Figure S1. SDS-PAGE gel showing the profile of SARS-CoV-2 Spike and RBD proteins under denaturing conditions. The electrophoresis was performed on a 10% (1 mm thick) gel at 160 V for 60 minutes in a Tris-Glycine-SDS running buffer, followed by Coomassie blue staining. A band of ~180 kDa, consistent with the full-length Spike protein, is observed in (A), and a band of ~38 kDa, corresponding to the RBD protein, is shown in (B). These results confirm the purity and integrity of the proteins used in this study. M: Molecular weight marker (Precision Plus Protein Standards, Bio-Rad) with values indicated on the left.

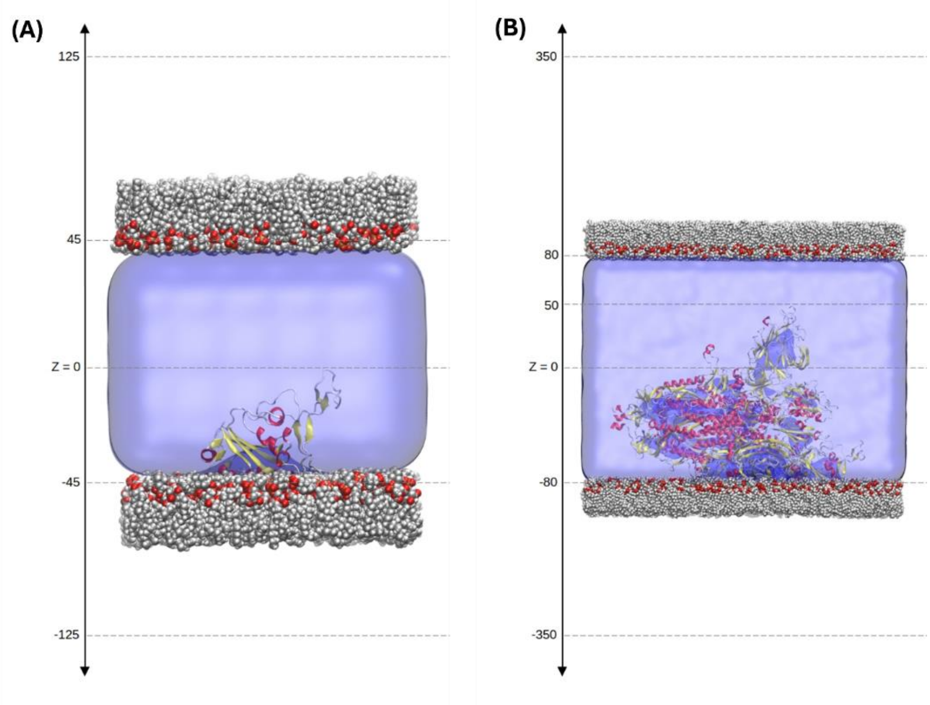

Figure S2. The initial configuration of the simulated periodic boxes is oriented along the lipid monolayer x-y axis and projected on the z-axis. Each system consists of two monolayers at different [Chol: PC] ratios in complex with SARS-CoV-2 RBD (A) or SARS-CoV-2 Spike (B). The membrane polar groups (colored red) are oriented inward, where they interact with water molecules (colored violet), and the aliphatic lipid tails (colored grey) face outward, exposing the nonpolar region to the vacuum (white areas). The periodic box extends along the z-axis, marking the boundaries of the periodic system. RBD and Spike are initially inserted in the bottom monolayer.

SAXS FITTING MODEL DATA

The scattering intensity (I(q)) is defined as I(q)∝ P(q). S(q), where P(q) represents the form factor and S(q) the structure factor. Both factors were fitted simultaneously, as described by Oliveira (*J. Appl. Cryst.* **2012**, *45*, 1278).

For the structure factor (S(q)), we employed the Caillé theory, represented by Equations 1 and 2 (A. Caillé, *C. R. Acad. Sci.* **1972**, *274B*, 891). The key parameters are D (lamellar periodicity), η (Caillé parameter), and the number of stacked layers in the lamellar phase. An example of the S(q) contribution is illustrated in Figure S3-A (blue dashed lines).

$$S(q) = 1 + 2\sum_{1}^{N-1}\left(1 - \frac{n}{N}\right)\cos(nqD)e^{-\frac{q^2}{2}\langle(u_n - u_0)^2\rangle} \quad \text{Eq. 1}$$

$$\langle(u_n - u_0)^2\rangle = \frac{h}{2p^2}[ln(pn) + g]D^2, n >> 1 \quad \text{Eq. 2}$$

Eletronic contrast=S4 Gaussians

$$r(z) = \sum_{n=1}^{4} a_n[G_s(z,z_n,\sigma_n) + G_s(z,-z_n,\sigma_n)]/(1+\delta_{i1}) \quad \text{Eq. 3}$$

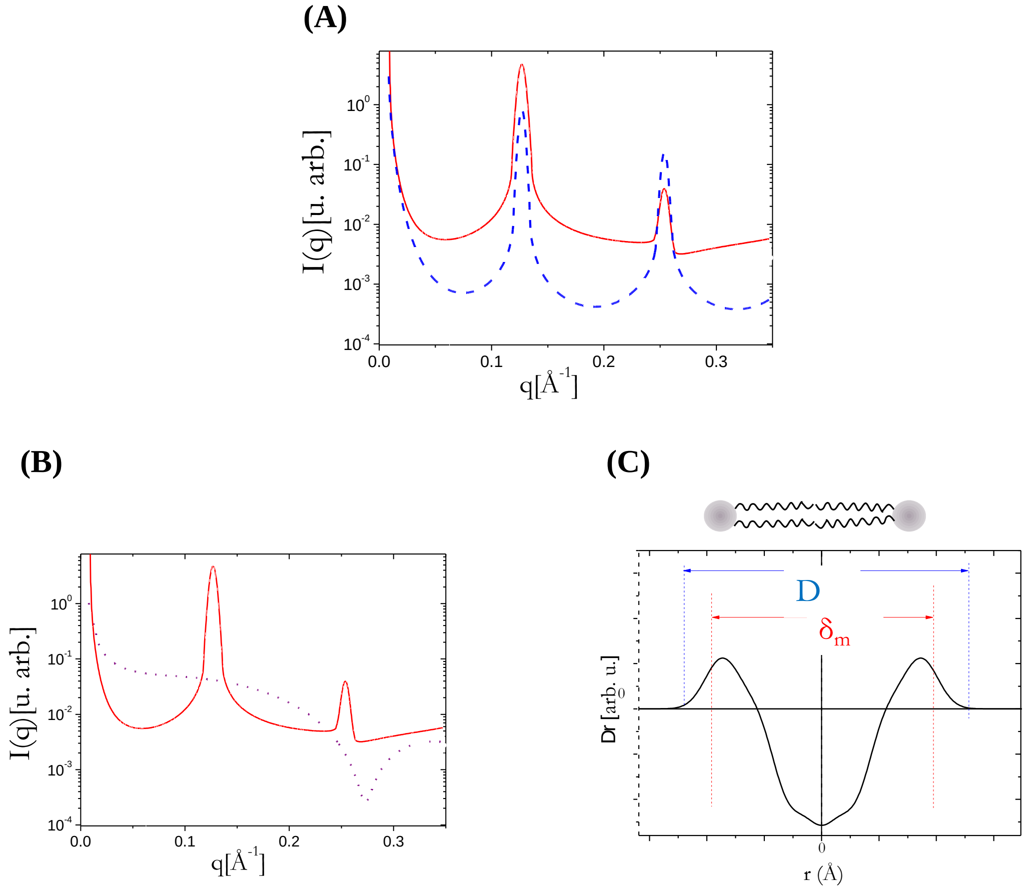

Figure S3. Examples of the SAXS data contributions: (A) Structure factor, (B) Form factor, and (C) Electronic contrast density (Δρ) of the PC membrane derived from the model.

We employed Gaussian deconvolution to model the bilayer form factor (*J. Appl. Cryst.* **2012**, *45*, 1278). This contribution is shown in Figure S3-B, which also presents an example of the electronic contrast density (Δρ) derived from the PC membrane (Figure S3-C).

Figures 1A and 1B in the manuscript primarily show form factor contributions, characterized by the absence of sharp peaks, indicating weak long-range interactions between layers. In contrast, Figures 1C and 1D demonstrate a significant influence of the structure factor on the scattering curves. The distinct "background" observed in Figures 1C and 1D arises from these structural contributions, which are less prominent in Figures 1A and 1B.

Additionally, we included a linear background (B1) in the fitting equation, as follows:

$$I(q) = \frac{S(q).P(q)}{q2} + Scale + B_1$$

This adjustment was made to improve the fit in the specified *q* range.

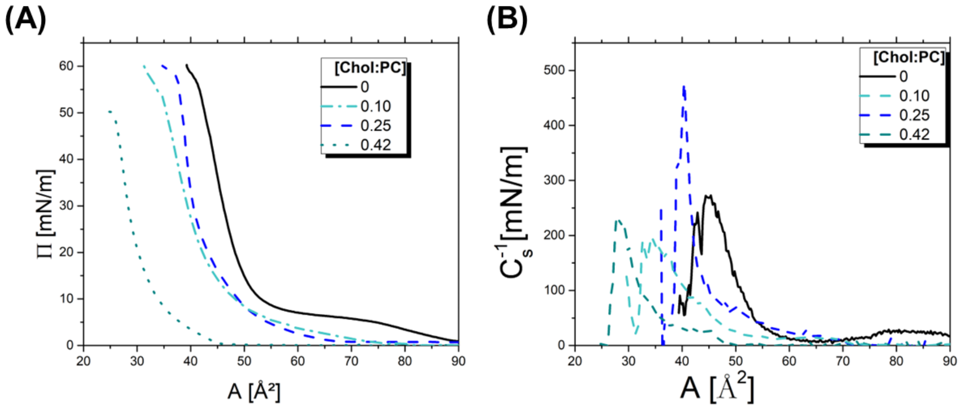

Figure S4. Surface pressure isotherm as a function of molecular area at different [Chol:PC] ratios (A) and the compressibility modulus (B) with the solid black line corresponding to PC and the blue dotted lines the variation in the molar ratio of cholesterol: 0; 0.10; 0.25 and 0.42.

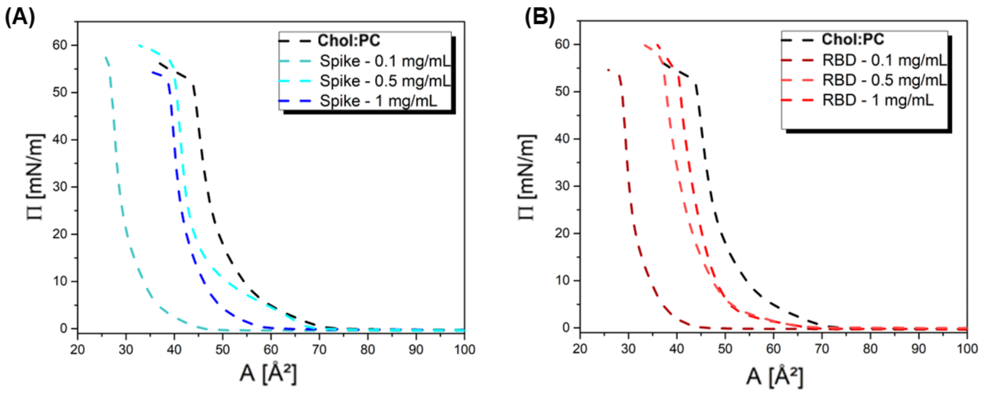

Figure S5. Surface pressure as a function of the molecular area in the presence of cholesterol, with the solid black line representing [Chol:PC] and the blue lines corresponding to monolayers with increasing concentrations of Spike protein (A), while the red lines represent monolayers with increasing concentrations of RBD protein (B).

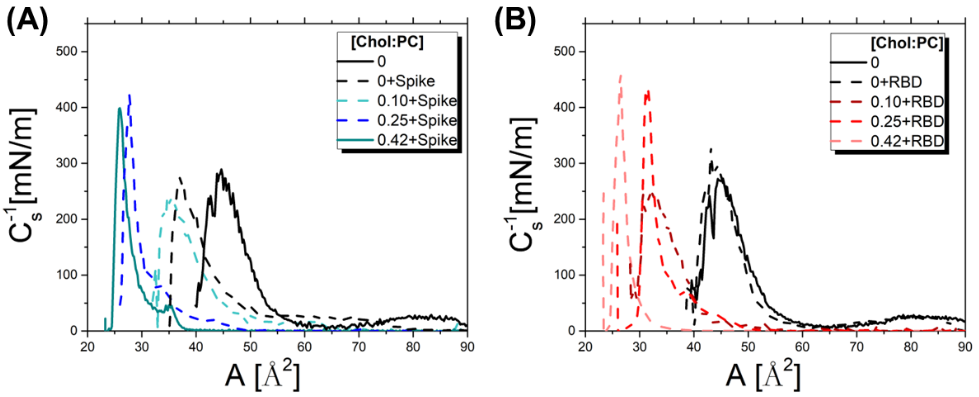

Figure S6. Compressibility modulus of the change in cholesterol concentration in the monolayer upon incorporating Spike proteins (A), represented by blue dashed lines, and RBD (B), represented by red dashed lines.

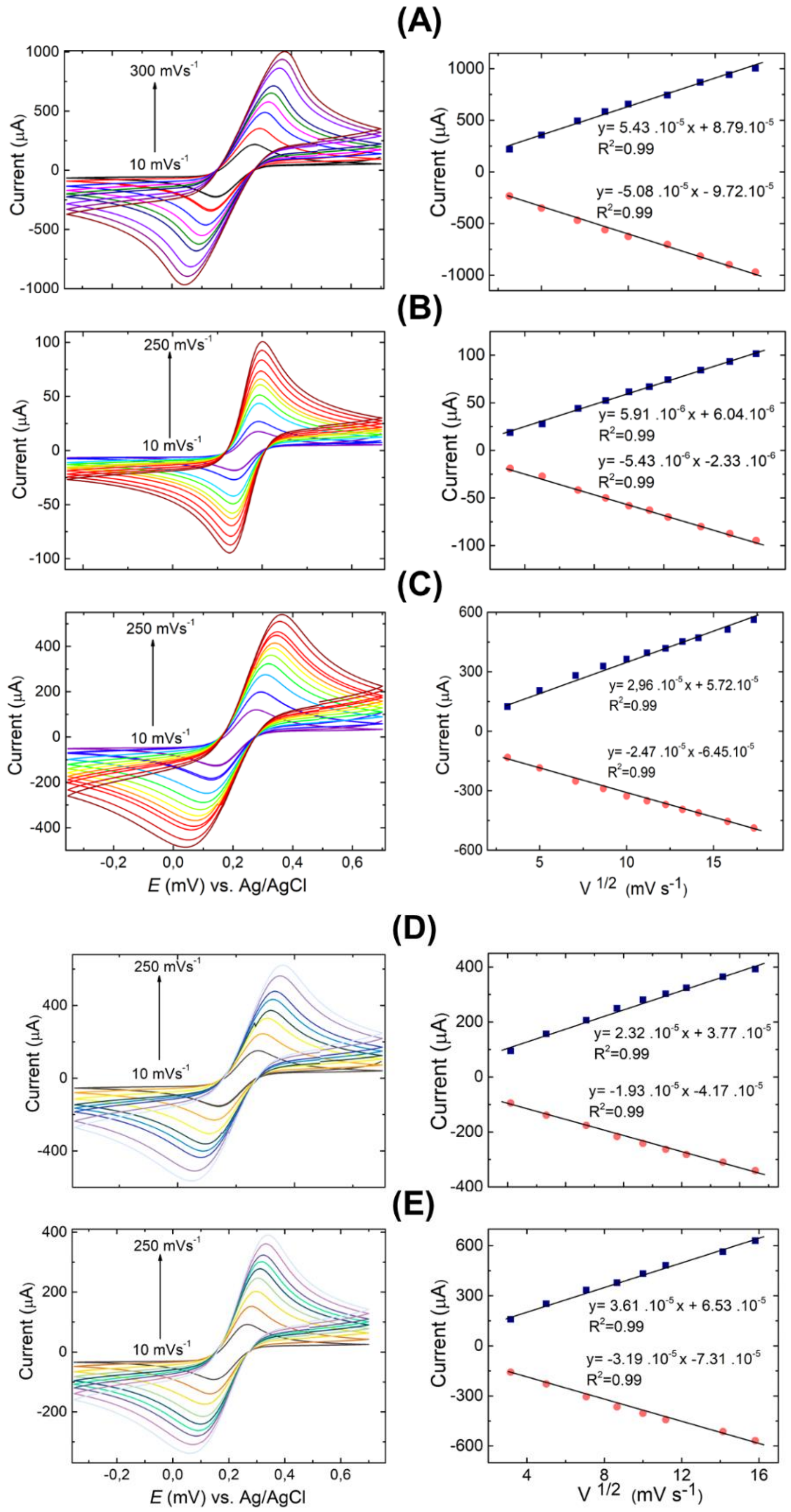

Figure S7. Electrochemical evaluation of ITO electrode surface modification in the presence of [Chol:PC] = 0.30 monolayer. Cyclic voltammograms of the previously cleaned ITO electrode at different scan rates and the corresponding plot of peak current versus the square root of the scan rate (A), and cyclic voltammograms of modified ITO electrodes with different proteins and concentrations, along with the respective plots of peak current versus the square root of the scan rate: PC:Chol + Spike (0.1 mg mL^{-1}) (B), PC:Chol + Spike (1 mg mL^{-1}) (C), PC:Chol + RBD (0.1 mg mL^{-1}) (D), and PC:Chol + RBD (1 mg mL^{-1}) (E). Electrolyte: 5 mmol L^{-1} $K_4Fe(CN)_6/K_3Fe(CN)_6$ in 0.1 mol L^{-1} KCl.

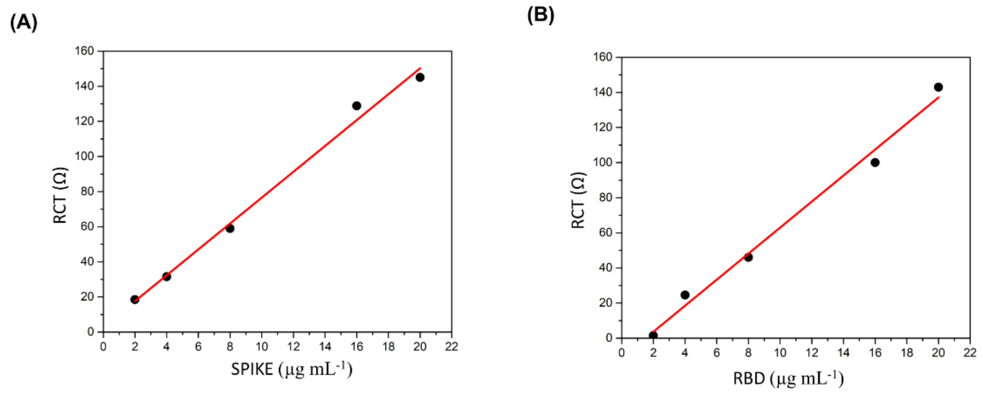

Figure S8. Calibration curve for Spike (A) and RBD (B) detection. These experiments were performed in triplicate, yielding detection limits for the Spike and RBD proteins of 364.1 ng mL^{-1} and 205.2 ng mL^{-1}, respectively.

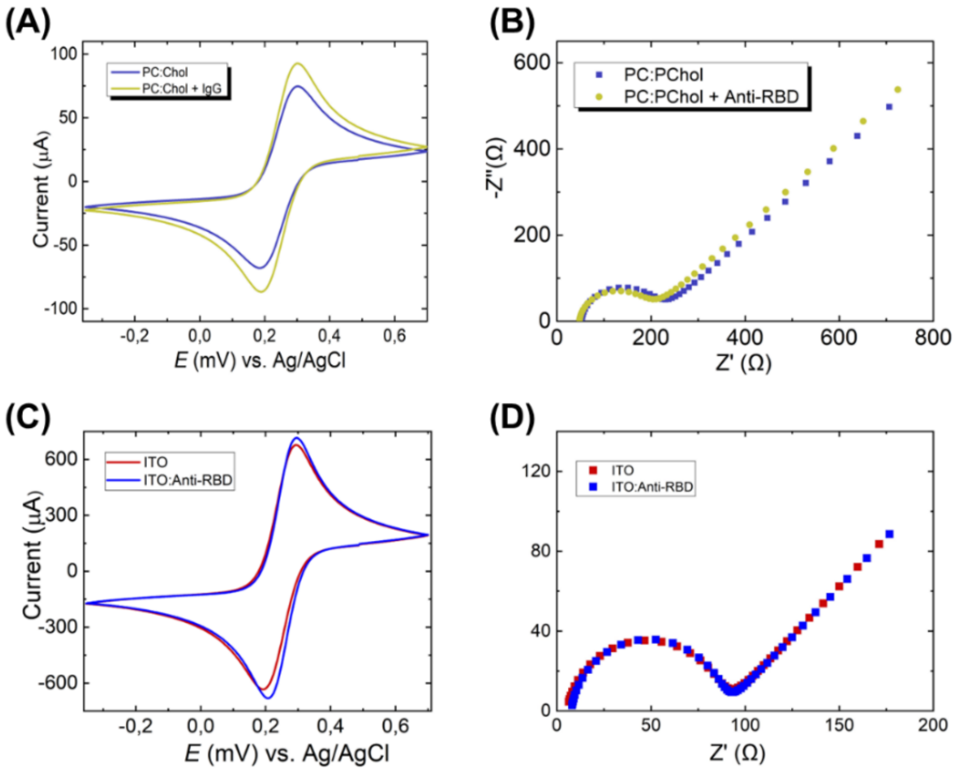

Figure S9. Electrochemical evaluation of ITO modification in [Chol:PC] = 0.25 monolayer in the presence of antiRBD antibody. Cyclic voltammetry (A) and Nyquist Diagram (B) for the pure [Chol:PC] monolayer, represented by the blue line, and in the presence of antiRBD antibodies, represented by the green line. Cyclic voltammetry (C) and Nyquist Diagram (D) for pure ITO, represented by the red line, and ITO in the presence of antiRBD antibody, represented by the purple line.

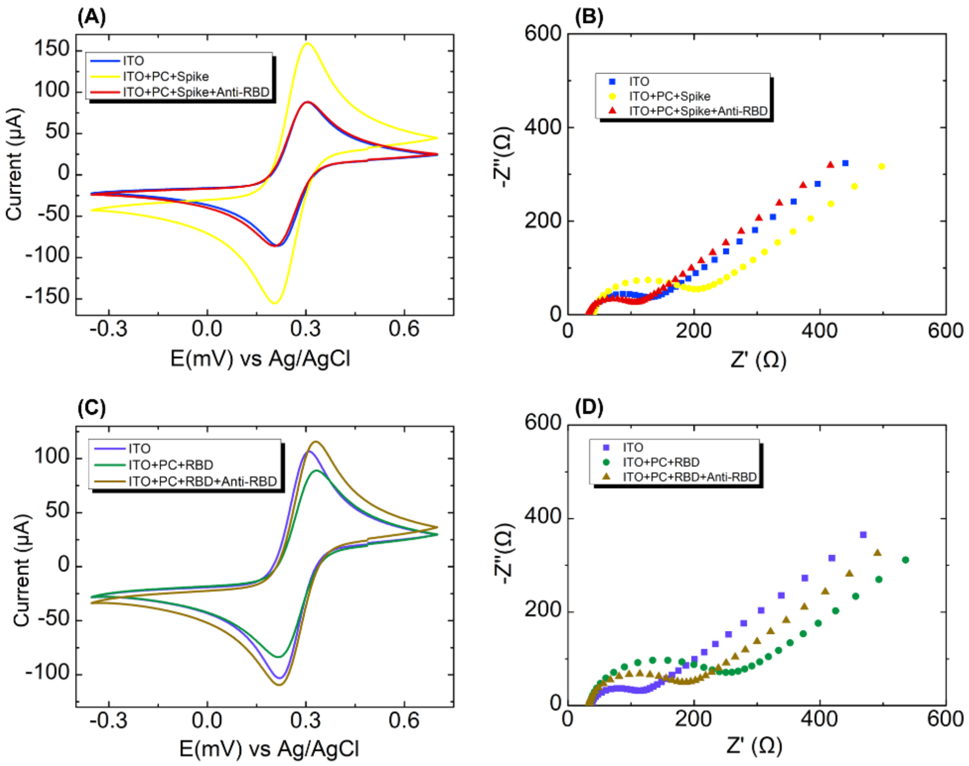

Figure S10. Electrochemical evaluation of ITO modification in [Chol:PC] = 0.25 monolayer with Spike and RBD in the presence of antiRBD antibody. Cyclic voltammetry (A) and Nyquist Diagram (B) for the monolayer with Spike, represented by the yellow line, and in the presence of antiRBD antibodies, represented by the red line. Cyclic voltammetry (C) and Nyquist Diagram (D) for the monolayer with RBD, represented by the green line, and in the presence of antiRBD antibody, represented by the brown line.

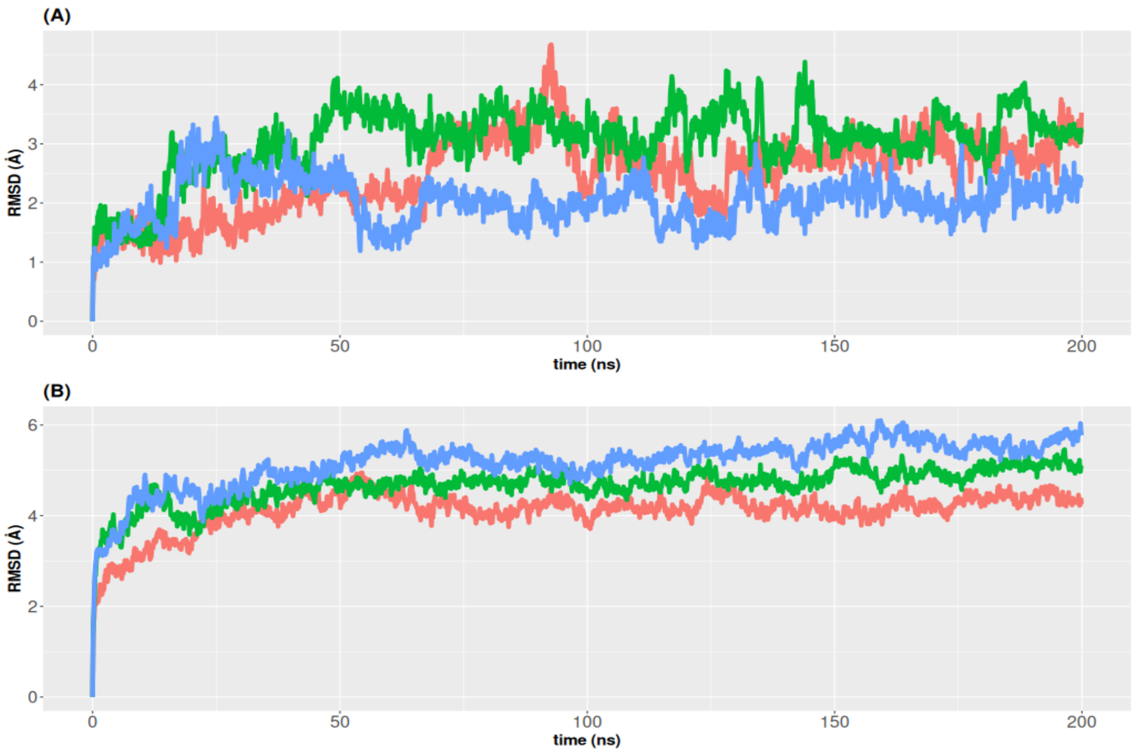

Figure S11. Root Mean Standard Deviations (RMSD) in Å for the [Chol:PC] lipid monolayers complexed with SARS-CoV-2 RBD (A) and SARS-CoV-2 Spike (B) over the MD trajectories. Red graphs: [Chol:PC] = 0, Green graphs: [Chol:PC] = 0.10, Blue graphs: [Chol:PC] = 0.30.

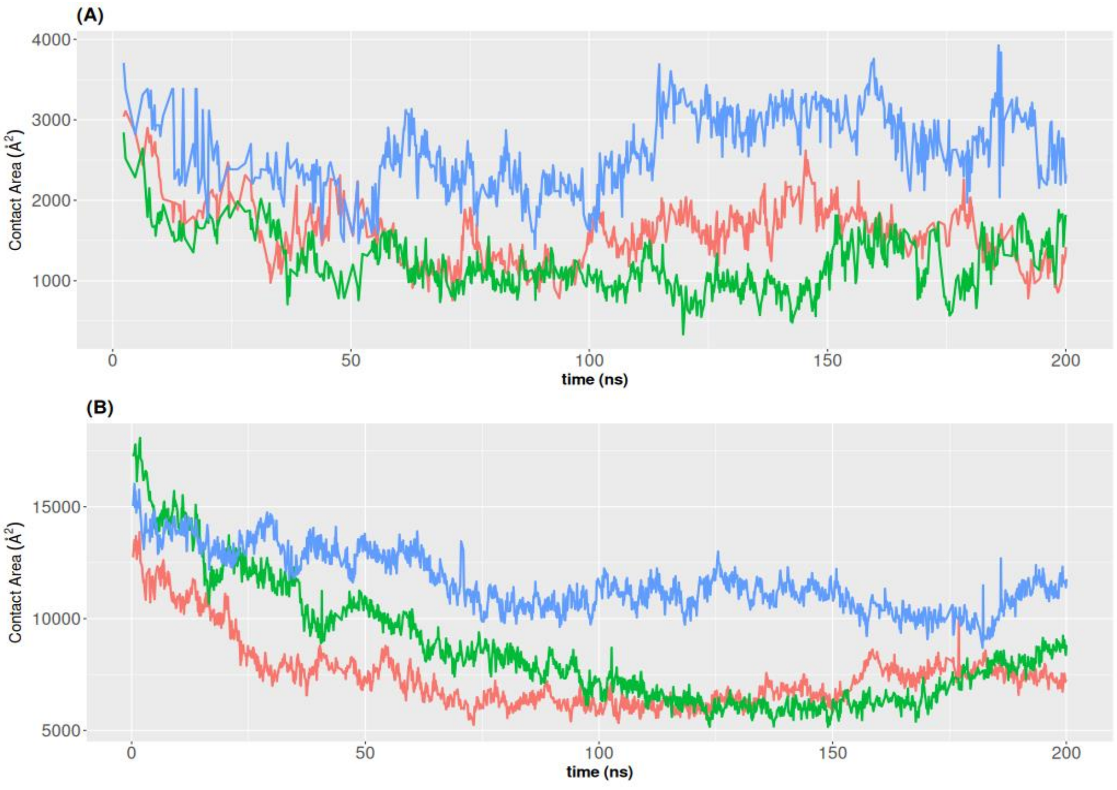

Figure S12. Time evolution of contact surface area (in Å^2) of SARS-CoV-2 RBD (A) and SARS-CoV-2 Spike (B) with the [Chol:PC] lipid monolayers over the MD trajectories. Red graphs: [Chol:PC] = 0, Green graphs: [Chol:PC] = 0.10, Blue graphs: [Chol:PC] = 0.30.

Equation S4. To measure the thickness of the lipid monolayers, phosphate groups aligned in the xy plane and projected onto the z-axis were used as a reference point, designated as MP1. At the lipid tail end, the terminal carbons of the chains were defined as the MP2 reference point. The instantaneous thickness is measured as:

$$Thickness(t) = \frac{1}{n_i}\sum_i Z_{MP1_i}(t) - \frac{1}{n_j}\sum_j Z_{MP2_j}(t)$$

where:

$Z_{MP1_i}(t)$ represents the z-axis Cartesian coordinates of the *i*-th phosphate group within the MP1 set at time *t*

$Z_{MP2_j}(t)$ represents the z-axis Cartesian coordinates of the *j*-th terminal carbon in the lipid tail (belonging to MP2) at time *t*

n_i is the number of phosphate groups in the MP1 set

n_j is the number of terminal carbons in the MP2 set

Equation S5. To measure the instantaneous protein depth (penetration) into the lipid monolayers, the following equation was used:

$$Depth(t) = \frac{1}{n_i}\sum_i Z_{MP1_i}(t) - \min(Z_{prot}(t))$$

where:

$Z_{MP1_i}(t)$ represents the z-axis Cartesian coordinates of the *i*-th phosphate group within the MP1 set at time *t*

$\min\left(Z_{prot}(t)\right)$ is the minimum z-axis Cartesian coordinate value observed for the protein at time *t* (excluding hydrogen atoms)

Equation S6.

$$I_p = (2.69 \times 10^5) n^{3/2} A D^{1/2} C V^{1/2}$$

where:

I_p is the peak current, n is the number of electrons transferred during oxidation or reduction, A is the electroactive area of the electrode (cm^2), D is the diffusion coefficient ($cm^2 s^{-1}$), C is the concentration of the electroactive species (mol/cm^3) and V is the scan rate ($V\ s^{-1}$). Through this equation we can find the electroactive area of the electrode using the solution of 5 mmol L^{-1} $K_4Fe(CN)_6/K_3Fe(CN)_6$ em 0.1 mol L^{-1} KCl.

Equation S7.

$$A = \frac{I_p}{V^{1/2}} \times \frac{1}{(2.69 \times 10^5) n^{3/2} D^{1/2} C}$$

For this equation the first term refers to the slope of the lines obtained from the graph of peak current versus the square root of the sweep rate.

where:

$n = 1, C = 5 \times 10^{-6}\, mol/cm^3$

The diffusion coefficient of potassium ferricyanide is equal to $6.39 \times 10^{-6}\, cm^2 s^{-1}$.
